# Optimizing Class G Cement for CO_2_ Storage (CCS): A Comprehensive Study on the Effects of Fly Ash and Eggshell Blends on Wellbore Cement Integrity

**DOI:** 10.1021/acsomega.5c12873

**Published:** 2026-05-30

**Authors:** Rockson Aluah, Adesina Fadairo

**Affiliations:** † 170404 University of North Dakota, Grand Forks, North Dakota 58202, United States; ‡ Oklahoma State University, Stillwater, Oklahoma 74078, United States

## Abstract

Carbon capture and storage (CCS) is a critical pathway for achieving net-zero emissions; however, maintaining long-term wellbore cement integrity under CO_2_-rich conditions remains a major technical challenge. Conventional Class G cement is highly vulnerable to carbonation–dissolution reactions, which increase porosity and permeability, weaken mechanical strength, and ultimately compromise zonal isolation. These degradation processes create potential leakage pathways that threaten storage security and reduce CO_2_ storage efficiency. This study presents a systematic optimization of Class G cement using fly ash (FA) and waste-derived eggshell powder (ESP) as supplementary cementitious materials. The objectives are to develop CO_2_-resistant formulations, quantitatively evaluate geomechanical, petrophysical, and geochemical performance under simulated downhole conditions, and identify the microstructural mechanisms responsible for enhanced durability. A novel formulation strategy is introduced, leveraging synergistic calcium–silica interactions between FA and ESP to fundamentally redesign the pore structure. Beyond conventional pore size reduction, this approach promotes pore network disconnection as the dominant sealing mechanism while simultaneously enabling a self-healing (autonomous sealing) response under CO_2_ exposure. The presence of reactive calcium phases from ESP enhances carbonate precipitation, allowing microcracks and pore throats to be progressively sealed through in situ mineralization. Cement systems [base cement (BS), FA/ESP (75%/25%), and FA/ESP (50%/50%)] were subjected to high-pressure, high-temperature aging (2000 psi, 170 °C) in CO_2_-saturated brine for up to 60 days. Comprehensive characterization was performed using Nuclear Magnetic Resonance, ultrasonic wave propagation (Auto-Lab 1500), X-ray diffraction, and Scanning Electron Microscopy. The FA/ESP (75%/25%) formulation exhibited superior performance, achieving a 75% reduction in permeability (0.01125 mD vs 0.045 mD for base cement) and an 82.22% decrease in porosity, while base cement showed a 56.7% increase. Mechanical properties improved significantly, with increases of 19.5% in Young’s modulus and 19.3% in Poisson’s ratio, indicating enhanced structural resilience. Mineralogical analysis revealed progressive calcite formation (46.4% to 56.5%), confirming active CO_2_ mineralization that transforms chemical degradation into a strengthening mechanism. These findings directly enhance wellbore integrity and CO_2_ storage efficiency by reducing permeability, minimizing leakage risk, and improving long-term containment. Pore network disconnection and improved mechanical strength ensure sustained zonal isolation, while CO_2_-driven mineralization enables self-sealing through calcite precipitation within pores and microfractures. The novel integration of industrial byproducts and agricultural waste offers a sustainable, cost-effective solution, establishing a new paradigm for durable, self-healing cement systems in geological carbon storage.

## Graphical abstract

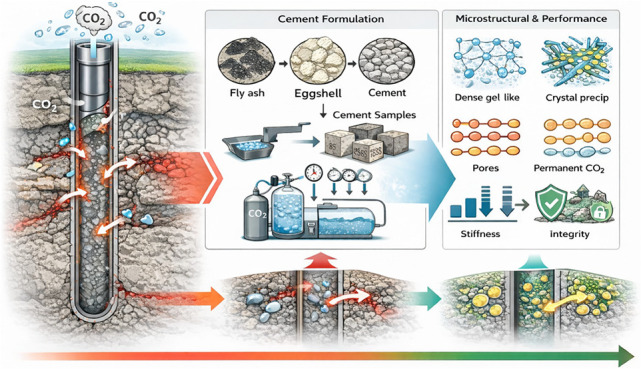

## 1 Introduction

Carbon capture and storage (CCS) has emerged as a critical technology for mitigating anthropogenic CO_2_ emissions and achieving global climate targets^1^ Geological sequestration in deep saline aquifers and depleted oil and gas reservoirs requires robust wellbore integrity over multidecadal time scales.^2^ Class G cement, the industry standard for primary cementing operations, plays a pivotal role in providing zonal isolation and preventing CO_2_ leakage.^3,4^ However, conventional cement formulations face severe degradation challenges when exposed to CO_2_-saturated environments at high temperature and pressure conditions typical of subsurface storage sites (Temperature: 90–170 °C, Pressure: 1000–3000 psi).^5–7^ The acidic environment created by dissolved CO_2_ leads to progressive mineral dissolution, increased porosity, compromised permeability barriers, and ultimately, loss of wellbore integrity, potentially undermining the long-term viability of CCS projects.^8,9^ Researchers^6,7^ have highlighted the potential for CO_2_ leakage through poorly sealed wellbores, emphasizing the need for resilient cement formulations (see in Figure 1).

**Figure 1:**
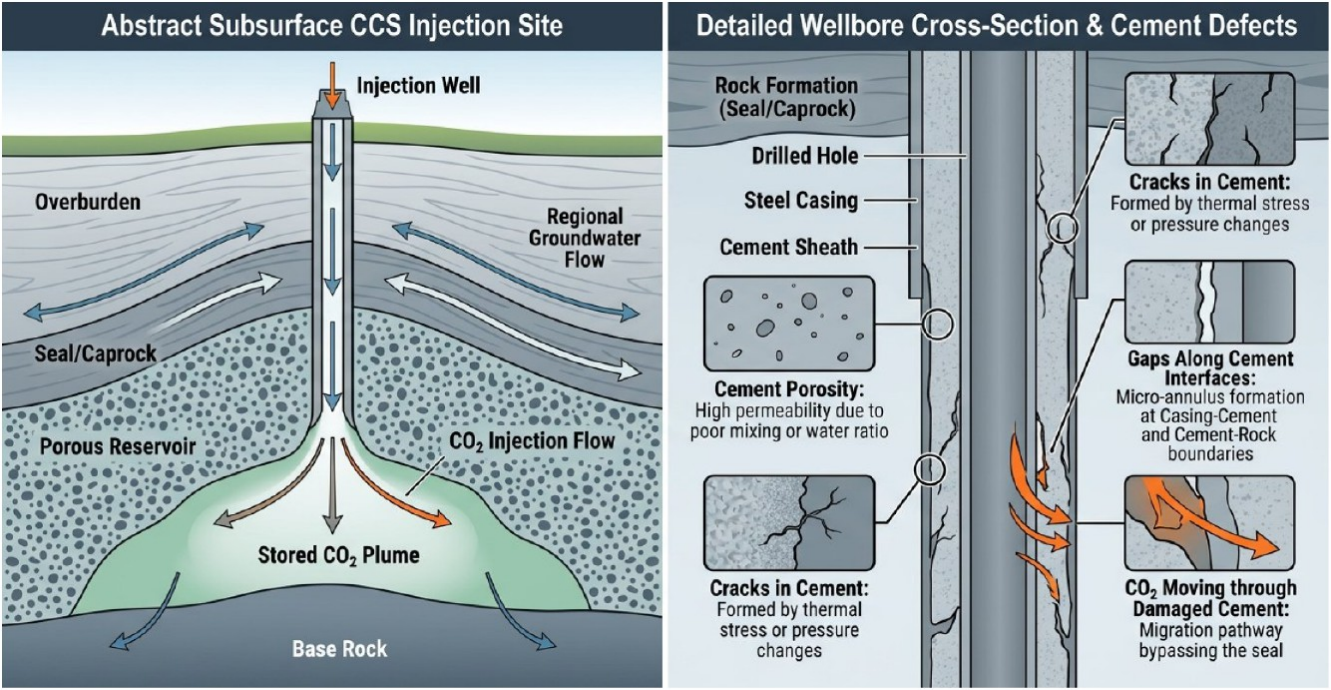
Demonstrates how improper cement design and formulation can create pathways for CO_2_ leakage, compromising the integrity and safety of CCS operations.

Recent advances in oil well cement modification have demonstrated that the strategic incorporation of supplementary cementitious materials and nanomaterials can significantly enhance cement performance under harsh downhole conditions. Experts^10^ investigated the magnetic field strength and temperature effects on oil well cement slurry modified with iron oxide nanoparticles, demonstrating that nanoparticle incorporation improved cement properties through enhanced particle packing and hydration kinetics, as quantified with Vipulanandan models. Their work showed that optimized nanoparticle concentrations (up to 2% by weight of cement) improved compressive strength by 15–20% at elevated temperatures (up to 80 °C), highlighting the potential of nanomaterial modification for deepwater applications. Similarly, Researcher,^11^ developed iron nanoparticle-modified smart cement for real-time monitoring of ultradeepwater oil well cementing applications, integrating sensing capabilities with mechanical enhancement. Their innovative approach demonstrated that functional additives could serve dual purposes: structural improvement and in situ property monitoring through piezoresistive behavior. Other nanomaterial studies have shown similar promise; for instance, nanosilica incorporation in Class G cement has been observed to improve compressive strength and reduce permeability,^12^ while self-healing cement systems incorporating microcapsules containing healing agents have demonstrated potential for autonomous crack repair in response to CO_2_-induced damage.^13^ These studies established important precedents for cement modification yet focused primarily on ambient CO_2_ conditions and did not address the specific challenges of long-term exposure to CO_2_-saturated brines at elevated pressures and temperatures characteristic of CCS applications.

The mechanical behavior and bonding strength of modified cements under aggressive conditions were extensively studied. Previous studies by,^14^ conducted experimental investigations and modeling of fracture behavior, mechanical properties, and bonding strength of oil well cement, developing predictive frameworks based on fracture mechanics. Their work emphasized that interfacial bonding and crack propagation resistance are critical parameters governing long-term cement integrity, particularly under cyclic loading and thermal-stress conditions. Building on this foundation,^15,72^ we developed novel moduli-based analyses for predicting the compressive strength of oil well cement slurries based on chemical composition at different temperature conditions. Their modeling approach demonstrated that the Ca/Si ratio, aluminate content, and sulfate balance significantly influence strength development kinetics and ultimate mechanical properties, with temperature playing a modulating role in hydration reactions. These studies provided valuable insights into cement behavior under thermal stress; however, they did not specifically address the unique degradation mechanisms induced by supercritical CO_2_ exposure, where chemical attack dominates over purely mechanical or thermal effects.

Despite these advances in cement modification and characterization, current approaches predominantly focus on reducing pore size through enhanced hydration, increased packing density, or pore-filling additives. While these strategies show promise for conventional oil well applications, they face fundamental limitations when applied to CO_2_ storage environments. The aggressive carbonation reactions typical of CO_2_-saturated systems can progressively dissolve calcium hydroxide (portlandite) and decalcify calcium-silicate-hydrate (C–S–H) gel, leading to increased porosity despite initial pore refinement efforts. Critically, even fine-pore networks remain vulnerable to CO_2_ permeation if pores remain interconnected, providing continuous diffusion pathways for corrosive fluids. This represents a fundamental paradigm gap in current cement design philosophy: the focus on pore size rather than pore-network connectivity. Furthermore, most previous studies on pozzolanic additives for cement modification have explored conventional materials (silica fume, metakaolin, and blast furnace slag) or expensive nanomaterials, with limited attention to sustainable, low-cost industrial waste materials specifically optimized for CO_2_ resistance. Fly ash (FA), an aluminosilicate byproduct of coal combustion, is a well-established pozzolanic material that reacts with calcium hydroxide to produce additional calcium-silicate-hydrate (C–S–H), thereby enhancing strength and durability.^16,17^ Studies have shown that fly ash incorporation improves pore structure, reduces permeability, and enhances resistance to CO_2_-induced degradation in cement systems.^9,18^ Various investigations have explored cement mixtures with different fly ash proportions (ranging from 16.7% to 50%), demonstrating correlations between carbonation depth and pozzolanic material content, as well as reductions in portlandite levels.^19^ Additionally, the combination of slag cement with fly ash has been shown to mitigate CaCO_3_ precipitation issues,^19^ while comparisons between lightweight and normal-weight fly ash-containing cements have demonstrated superior durability and permeability performance for certain formulations.^20^ Beyond conventional fly ash applications, geopolymer cement systems based entirely on alkali-activated fly ash have shown promise, with Class F fly ash-based geopolymers exhibiting higher compressive and shear strength, improved durability in acidic environments, and lower chemical shrinkage compared to Portland cement.^21–24^ More recently, Class C fly ash-based geopolymers have been successfully formulated and tested, showing enhanced rheological behavior and potential applicability in plug and abandonment operations.^25–27^ Eggshell powder (ESP), a calcium-rich agricultural waste that is composed primarily of calcium carbonate, represents another promising additive. Research has indicated that ESP can accelerate cement hydration, increase compressive strength, and decrease porosity,^28^ with additional studies suggesting antibacterial properties due to CaO-derived reactive oxygen species.^29^ However, the synergistic integration and ratio optimization of FA and ESP specifically for CCS conditions (high pressure, high temperature, CO_2_-saturated brine) remain largely unexplored. Moreover, the mechanism by which such blended systems achieve CO_2_ resistance—whether through simple pore filling, beneficial carbonation, or more fundamental alterations to pore network topology—has not been adequately elucidated.

The present study addresses these critical knowledge gaps by introducing a novel paradigm in cement design for CO_2_ storage applications: strategic elimination of pore network connectivity rather than mere pore size reduction. We hypothesize that optimized blending of fly ash and eggshell powder at specific ratios can create a disconnected pore network architecture that prevents CO_2_ percolation even in the presence of residual porosity. This approach represents a fundamental departure from conventional strategies and offers potential advantages for long-term CO_2_ storage integrity. Specifically, this research advances the state of the art in three key aspects: first, we systematically optimize FA/ESP blend ratios (50%/50% vs 75%/25%) specifically for CO_2_ resistance under realistic CCS conditions (2000 psi, 170 °C, CO_2_-saturated brine), moving beyond ambient condition studies. Unlike previous nanoparticle modifications^10,11^ that require expensive materials and complex processing, our approach leverages abundant industrial and agricultural wastes, offering both economic and environmental benefits. Second, we employ comprehensive multiscale characterization, including nuclear magnetic resonance (NMR), permeability, ultrasonic wave testing, X-ray diffraction (XRD), and scanning electron microscopy (SEM), to elucidate the mechanisms underlying superior CO_2_ resistance. This integrated approach extends beyond the mechanical property focus of previous studies^14,15^ to directly quantify pore network evolution, connectivity changes, and mineralogical transformations over extended exposure periods (10, 30, and 60 days). Our mechanistic investigation reveals that pore disconnection, not merely pore refinement, is the critical factor governing CO_2_ resistance. Third, we demonstrate unprecedented performance improvements: 75% permeability reduction, 82.22% porosity decrease, and a 19.5% increase in Young’s modulus that significantly exceed previously reported enhancements for pozzolanic systems. More importantly, we show that beneficial carbonation (21.8% calcite increase) occurs simultaneously with permeability reduction, indicating active CO_2_ mineralization rather than passive degradation. This dual benefit—improved sealing and permanent CO_2_ fixation—has not been previously achieved in cement systems.

### 1.1 Chemical Reactions Between CO_2_-Saturated Brine and Cement

Class G Portland cement is primarily composed of four main components: 50% tricalcium silicate (C_3_S), 30% dicalcium silicate (C_2_S), 12% tetracalcium aluminoferrite (C_4_AF), and 5% tricalcium aluminate (C_3_A), as reported by ref.^20^ When hydrated, the cement forms predominantly C–S–H gel (70%) and calcium hydroxide (15–20%), with C–S–H serving as the primary binding agent in the hydrated cement.^20^ Table 1 provides an overview of the various experimental configurations employed by different researchers to investigate how cement is altered when exposed to fluids containing CO_2_. The carbonation process of Portland cement, which has been extensively studied, can be summarized as follows:

**Table 1:** Summary the Various Experiments and Conditions by Different Researchers to Examine the Effects of CO_2_-rich Environments on Cement Integrity^a^

Reference	Curing	Method	T (°C)	P (MPa)	Sample (mm)	Fluid	Time	Main Findings
Kutchko et al., 2007;^20^ Fentaw JW et al., 2024 ^31^	28 d	Static batch	50	30.3	12 × 130	scCO_2_, CO_2_-brine	9 d	Carbonation zones observed; permeability decreased
Shiferaw et al., 2019 ^32^	28 d	Hydration (EP)	20	Atm	N/S	Water	28 d	EP accelerated hydration but less effective than limestone
Duguid and Scherer, 2010 ^3^	28 d	Flow-through	20, 50	Atm	7.5 × 200	CO_2_-brine	≤6 mo	Higher temperature increased degradation rate
Barlet-Gouédard et al., 2009 ^33^	72 h	Static batch	90	20.7	25 × 50	scCO_2_, CO_2_-water	≤6 mo	Rapid initial carbonation followed by slower alteration
Abd Rahman et al., 2020 ^34^	7 d	Water bath	20	Atm	50 × 100	Water	28 d	Geopolymer cement showed improved expansion
Liteanu and Spiers, 2011 ^35^	40 d	Triaxial test	80	40	36 × 72	scCO_2_, CO_2_-brine	30 d	Initial strength gain followed by degradation
Carey et al., 2007 ^5^	Field (30 yr)	Core analysis	54	18	Field core	CO_2_-rich fluids	30 yr	Extensive carbonation with maintained integrity

a Note: d = days; h = hours; yr = years; mo = months; *T* = temperature; *P* = pressure; atm = atmospheric pressure; scCO_2_ = supercritical carbon dioxide; CO_2_-brine = CO_2_-saturated brine; EP = ettringite promoter; N/S = not specified.

In carbon dioxide storage scenarios, the injected CO_2_ combines with water, forming carbonic acid (as shown in eq 1). This process results in a reduction of the pH level in Portland cement, which typically maintains a pH above 12.5 under normal conditions. The resulting pH imbalance prompts the carbonic acid to diffuse into the cement matrix, seeking equilibrium. This diffusion process can have significant implications for the cement’s long-term integrity and performance in CO_2_ storage environments.

1 $${\mathrm{CO}}_{2}\left(g\right){+H}_{2}O\left(l\right)\to H_{2}{\mathrm{CO}}_{3}\left(\mathrm{aq}\right)$$

The carbonic acid interacts with calcium hydroxide (Ca­(OH)_2_(s)) present in the cement, as illustrated in eq 2, resulting in the formation of calcium carbonate (CaC*O*_3_(*s*)). The molar volume of Ca­(OH)_2_ is 33.1 cm^3^ /mol, while CaCO_3_ occupies 36.9 cm^3^ /mol, representing an approximately 11.5% volume increase. This volume expansion contributes to reduced porosity and permeability but can also generate internal stress in the cement matrix, potentially leading to microcracking if not properly accommodated by the cement’s microstructure. The increased volume can generate significant internal stress within the cement matrix, potentially causing the development of microcracks. These structural changes can have important implications for the cement’s integrity and long-term performance in CO_2_ storage applications.

2 $$\mathrm{Ca}{\left(\mathrm{OH}\right)}_{2}\left(s\right){+H}_{2}{\mathrm{CO}}_{3}\left(\mathrm{aq}\right)\to {\mathrm{CaCO}}_{3}\left(s\right){+2H}_{2}O\left(1\right)$$

Extended exposure to carbonic acid initiates a secondary reaction, where the previously formed calcium carbonate dissolves, producing calcium bicarbonate. This compound is highly soluble and can readily leach out of the cement matrix, as shown in eq 3. Following this, the carbonic acid begins to break down the calcium-silicate-hydrate (C–S–H) gel, a primary component of cement’s strength. This degradation results in the formation of amorphous silica, which lacks structural integrity and is characterized by high porosity. As described in eq 4, this process leads to a significant increase in the cement’s porosity and permeability, while simultaneously causing a marked decrease in its strength. These changes can severely compromise the cement’s ability to maintain an effective seal in CO_2_ storage environments over extended periods.

3 $${\mathrm{CaCO}}_{3}\left(s\right){+H}_{2}{\mathrm{CO}}_{3}\left(\mathrm{aq}\right)\to \mathrm{Ca}{\left({\mathrm{HCO}}_{3}\right)}_{2}$$

4 $${\mathrm{C‐S‐H and}/\mathrm{or crystalline phases}+H}_{2}{\mathrm{CO}}_{3}\left(\mathrm{aq}\right)\to {\mathrm{SiO}}_{2}\left(\mathrm{gel}\right){+\mathrm{CaCO}}_{3}\left(s\right){+H}_{2}O\left(l\right)$$

Early age/accelerated carbonation often increases strength and decreases porosity through pore-filling calcite formation. Late-stage carbonation typically reduces strength via C–S–H decalcification, increasing porosity. In our FA/ESP system, CO_2_ exposure enhanced properties (porosity −82%, permeability −75%) through optimized calcite precipitation and pore network disconnection, demonstrating carbonation’s condition-dependent outcomes. This combination of effects raises concerns about potential gas migration through the cement matrix. To enhance cement’s resistance to CO_2_, two main strategies can be employed: (1) decreasing the portlandite (Ca­(OH)_2_) content and modifying the C–S–H structure, or (2) lowering the water-to-cement ratio to reduce porosity and permeability. Pozzolans are commonly introduced to achieve the first strategy by reducing portlandite content. These materials primarily react with Ca­(OH)_2_ to form secondary C–S–H.^12,30^ Fly ash, a waste product from coal combustion, is one of the most widely used pozzolans for this purpose. Composed mainly of alumina and silica, fly ash undergoes a geopolymerization process when combined with an alkaline solution. This reaction transforms fly ash into a binder, predominantly an aluminosilicate gel, which behaves like cement when mixed with water. The use of fly ash-based cement offers additional benefits besides enhancing CO_2_ resistance. It provides an environmentally friendly alternative to traditional cement, as it repurposes waste material. Furthermore, fly ash-based cement is often more cost-effective than conventional options, making it an attractive choice for various construction and well-cementing applications.

The specific objectives of this research are: (1) to systematically evaluate the CO_2_ resistance of Class G cement modified with fly ash/eggshell powder blends at two ratios (50%/50% and 75%/25%) under simulated CCS conditions (2000 psi, 170 °C, CO_2_-saturated brine) over 60 days, benchmarked against unmodified BS; (2) to comprehensively characterize the evolution of pore structure (porosity, pore size distribution, connectivity), transport properties (permeability), mechanical properties (Young’s modulus, Poisson’s ratio), and mineralogical composition (XRD phase analysis) as functions of FA/ESP ratio and exposure duration; (3) to elucidate the fundamental mechanisms by which optimized FA/ESP blends achieve superior CO_2_ resistance, with particular focus on distinguishing between pore size reduction effects and pore network disconnection phenomena through integrated NMR, permeability, and microstructural analyses; (4) to establish the pore disconnection paradigm as a novel design principle for CO_2_-resistant cement systems, demonstrating that strategic elimination of percolation pathways, rather than mere pore refinement, governs long-term wellbore integrity in CCS applications; and (5) to quantify the dual benefits of active CO_2_ mineralization and enhanced sealing performance, demonstrating that beneficial carbonation can occur simultaneously with permeability reduction through optimized Ca–Si ratio control. By addressing these objectives, this study provides both fundamental insights into cement-CO_2_ interaction mechanisms and practical formulation guidelines for next-generation CCS wellbore materials, advancing the field toward sustainable, long-lasting carbon storage infrastructure.

## 2 Methodology

### 2.1 Materials

The materials used in this study include Class G cement, defoamer, fluid loss additive, retarder, water, eggshell powder, Class C fly ash, and silica solution, which was sourced from Nouryon Pulp and Performance Chemicals Inc. in the United States.

Portland cement: Class G cement, sourced from Dyckerhoff Company, was used in this research.

Fly ash: Fly ash sourced from a North Dakota coal powder plant station was used in this study. Table 2 shows the elemental composition of fly ash measured using X-ray fluorescence (XRF).

**Table 2:** XRF Analysis for Chemical Composition of Eggshell Powder and Fly ash^12,38^

Component	Fly ash Mass (%)	Eggshell powder Mass (%)	Class G Cement (%)
Fe_2_O_3_	11.21	–	4.34
TiO_2_	–	–	-
MgO	5.14	1.12	1.43
SiO_2_	24.54	0.05	21.43
Al_2_O_3_	10.96	0.05	3.18
MnO	–	–	1.43
SO_3_	10.68	0.49	2.92
CaO	17.74	98	64.48
Na_2_O	15.58	-	0.13
K_2_O	15.84	-	0.34
P_2_O_5_	–	0.1	-
LOI	-	-	1.08

Eggshell powder: Eggshells obtained from university restaurants and apartments were used in this study. Following the same procedure described in the literature,^36^ the eggshells were thoroughly washed with tap water to remove any contaminants, with special attention given to removing the egg membranes. This cleaning step is crucial to ensure the purity of the final product. The shells were then oven-dried at 90 °C for 1 h, removing all moisture that could interfere with grinding and later cement hydration processes. Once dried, the eggshells were ground into a fine powder. This grinding process is critical as it increases the surface area of the eggshell particles, potentially enhancing their reactivity in cement mixtures. The ground powder was then sieved through a 20 μm mesh, adhering to the specifications of^12^ Table 2 shows the elemental composition of eggshell powder measured using X-ray fluorescence (XRF).

Ilmenite (FeTiO_3_) at 36.3% BWOC serves primarily as a weighting agent (*ρ* = 4.5–5.1 g/cm^3^) to achieve target slurry density.^36^ As a relatively inert iron–titanium oxide with low solubility at alkaline pH, ilmenite minimally participates in hydration reactions. Its inclusion creates a dilution effect, reducing reactive cement phases by ∼27% (36.3%/136.3% total), which decreases available portlandite and C–S–H for CO_2_-induced degradation while also reducing baseline mechanical properties.^36,37^

### 2.2 Preparation of Formulated Cement Slurry

The cement slurry was prepared following API RP 10B guidelines as recommended by the American Petroleum Institute. Table 3 details the composition of each cement mixture. The process began with accurately weighing and uniformly mixing all dry ingredients (Class G cement, fly ash, and eggshell powder) before incorporating additives. Using a water-to-cement ratio of 0.44 by mass (44% BWOC—by weight of cement, where “cement” includes Class G Portland cement plus all supplementary cementitious materials), distilled water (ASTM Type II) was placed in a sturdy mixing container. The water temperature was maintained at 23 ± 2 °C throughout the mixing process. A Festool 575213 MX 1200 EEF mixer, running at 1000 rpm, was used to blend liquid additives (defoamer and silica solution) first. Solid additives (fluid loss additive, retarder, and dispersant) were premixed with a small amount of distilled water to ensure homogeneity before being added to the mixture. The dry powder blend (cement, fly ash, and eggshell powder) was then gradually and evenly introduced into the container with fluid additives while the mixer continued at 1000 rpm. Slow addition of the dry powder was advised to prevent clumping and ensure uniform dispersion. The mixer was then operated at 1000 rpm for 2 min to achieve a smooth, homogeneous cement slurry free of lumps or air pockets. The prepared slurries were immediately transferred to specimen molds to prevent setting. All mixing was performed in a climate-controlled laboratory to ensure consistent slurry properties across all batches.

**Table 3:** Optimize Cement Slurry Formulations

Components	Base Cement Slurry	FA/ESP (75/25)	FA/ESP (50/50)
Cement (% BWOC)	100	100	100
Ilmenite (%)	36.3	36.3	36.3
Silica solution	35	35	35
Water (% BWOC)	44	44	44
Defoamer (% BWOC)	7	7	7
Dispersant (% BWOC)	0.25	0.25	0.25
Fluid Loss (% BWOC)	0.5	0.5	0.5
Retarder (% BWOC)	1.5	1.5	1.5
Eggshell Powder (% BWOC)	0	75	50
Fly Ash (% BWOC)	0	25	50

### 2.3 Preparation of the Cement Samples

Cement slurry was cast into 2 × 2 x 2 in^3^ cubic molds. Samples underwent initial curing at 100% humidity for 24 h, adhering to ASTM C1107 standards (ASTM C1107–20, 2020). Further curing occurred in a core flooding accumulator containing produced water (brine) under high-temperature, high-pressure (HTHP) conditions of 170 °C and 2000 psi for 72 h to simulate reservoir conditions. The cubes were subsequently drilled and cut to form cylindrical samples 2 in. in length and 1 in. in diameter at the UND Geology and Geological department. Sample surfaces were polished to ensure smoothness and evenness. Precise dimensions of the core plugs are presented in Table 4. Following drilling and cutting, the samples were oven-dried at 93 °C to eliminate moisture, and their dry bulk density was determined. Prepared samples (BS, FA/ESP (75/25), FA/ESP (50/50)) were submerged in brine within the core flooding accumulator. Pure CO_2_ was injected at 2000 psi and 170 °C for durations of 10, 30, and 60 days to mimic reservoir conditions and analyze the impact of CO_2_ exposure on wellbore cement integrity for carbon capture and storage (CCS) applications. For Nuclear Magnetic Resonance (NMR) porosity measurements, samples were vacuum-treated for 2 h to remove trapped air and moisture from pore spaces. They were then immersed in distilled water for 72 h to achieve full pore network saturation. To enhance saturation further, samples were placed in a core flooding accumulator at 2000 psi for 8 days prior to NMR analysis. For permeability measurements, core samples were cleaned using the Dean–Stark apparatus with toluene and ethanol solvents to remove residual pore fluids. Samples were then oven-dried at 93 °C for 48 h and weighed to obtain the dry weight (*W*_d_). The flowchart outlining the complete experimental workflow, including sample preparation, CO_2_ exposure, and analytical characterization procedures Figure 2. Finally, the cement samples were placed in a core container and subjected to vacuum conditions for 24 h to ensure complete air removal from pore spaces.

**Figure 2:**
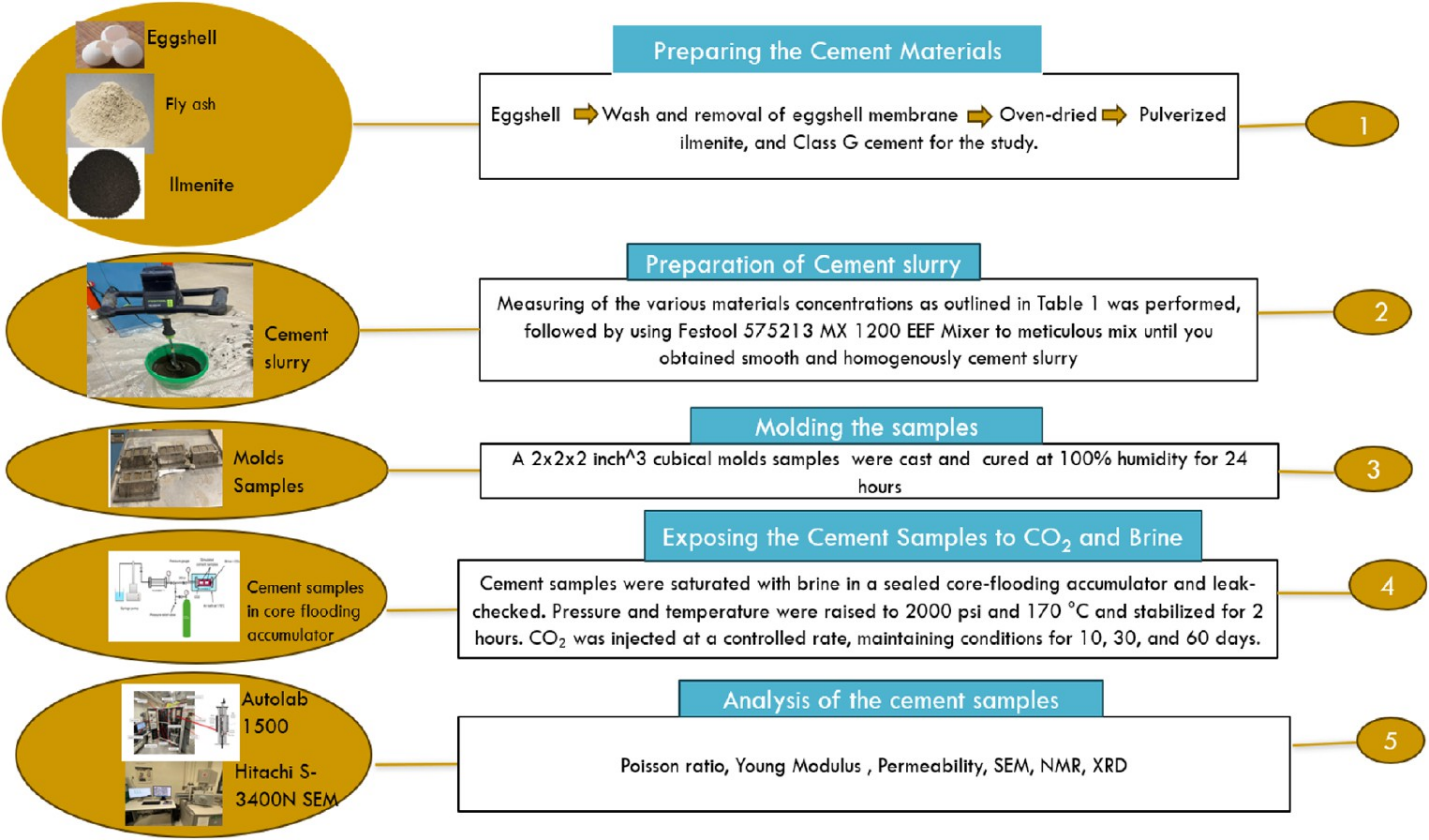
Flowchart outlining the methodology from sample preparation through analysis.

**Table 4:** XRD Mineralogy Composition Analysis of Cement Samples Exposed to CO_2_ Under Simulated Downhole Conditions Over Time^a^

Sample	Mineral Phase	10 Days (wt %)	30 Days (wt %)	60 Days (wt %)
BS	Portlandite	27.4	30.0	30.0
	Coesite	11.1	10.1	10.1
	Alite	10.0	9.5	9.0
	Brownmillerite	9.1	8.8	12.8
	Halite	6.5	7.5	2.5
	Larnite	10.2	9.2	8.2
	Pyroaurite	8.0	9.0	8.0
	Sodium chlorate	4.3	4.4	2.4
	Gismondine	9.0	8.0	8.5
	Magnetite	4.3	3.5	2.5
FA/ESP (75/25)	Calcite	46.4	50.5	56.5
	Brownmillerite	13.0	12.0	14.0
	Hydrocalumite	16.9	11.4	10.9
	Larnite	8.0	8.0	6.0
	Halite	7.0	7.0	4.0
	Gismondine	2.4	2.4	2.4
	Pyroaurite	3.9	3.9	3.9
	Maghemite	0.1	–	–
	Portlandite	0.8	2.2	3.5
	Alite	1.5	2.3	3.5
FA/ESP (50/50)	Calcite	30.4	26.4	32.0
	Brownmillerite	23.0	25.0	23.0
	Hydrocalumite	14.9	14.9	14.9
	Larnite	10.0	12.8	10.0
	Halite	7.0	8.0	9.0
	Gismondine	2.4	5.4	2.4
	Pyroaurite	3.9	3.0	3.9
	Maghemite	0.1	0.2	0.1
	Portlandite	4.2	3.3	2.3
	Alite	5.0	2.0	3.3

a Note: wt % = weight percentage; “–” indicates phase not det.

### 2.4 Procedure for Exposing Cement Formulation Samples to CO_2_ and Brine

1 The cement samples were placed in the core flooding accumulator and filled with the produced water (brine), ensuring full saturation of the samples. The accumulator was sealed and checked for leaks, as shown in Figure 3.
2 The pressure was gradually increased in the accumulator to the target pressure of 2000 psi, for typical CO_2_ storage conditions. The temperature was adjusted to the desired level of 170 °C to simulate downhole conditions. The system was allowed to stabilize for 2 h.
3 The accumulator was pressurized with CO_2_ at a controlled injection rate, with pressure and temperature maintained at 2000 psi (13.8 MPa) and 170 °C, respectively, throughout the exposure period.
4 The pressure of 2000 psi and temperature of 170 °C were kept constant in the CO_2_-brine-cement system for the following durations: 10 days, 30 days, and 60 days.
5 After each exposure period, the accumulator was slowly depressurized, and the corresponding set of samples was carefully removed. The mass and dimensions were recorded, and the visual appearance of each sample was observed.

**Figure 3:**
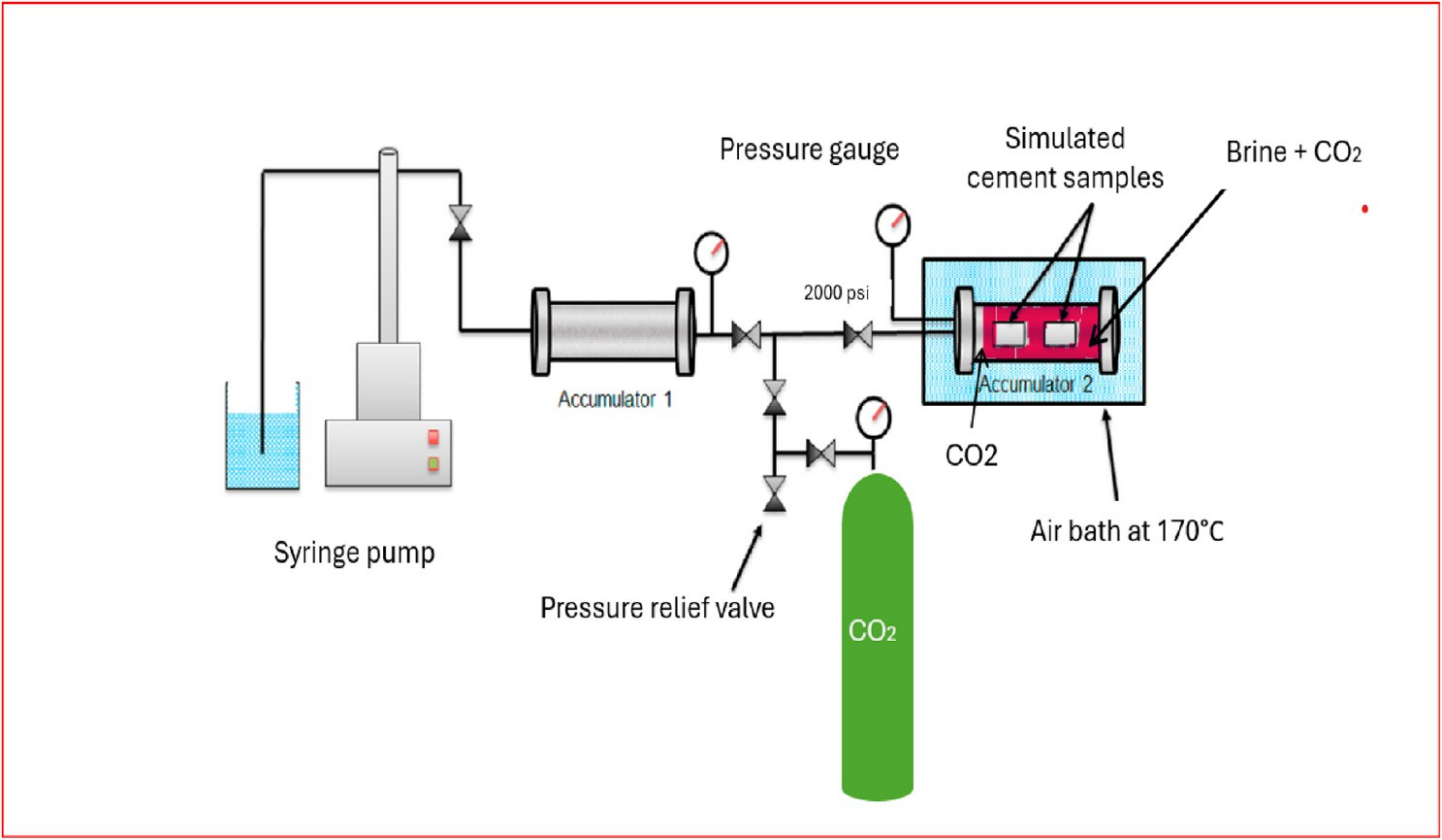
Schematic of high-pressure, high-temperature CO_2_ exposure setup for cement sample testing in simulated downhole conditions.

### 2.5 Petrophysical Properties Measurement

Porosity and permeability are critical properties in assessing wellbore integrity, particularly for CO_2_ storage and long-term subsurface applications.^39^ Permeability (K) of the cement samples was evaluated using the New England Research (NER) AutoLab 1500 at the Department of North Dakota, as shown in Figure 4. This apparatus utilizes a pulse-decay method, injecting nitrogen gas to assess permeability under conditions mimicking those in CO_2_ storage reservoirs. The cement samples, encased in a holder, are subjected to controlled confining and pore pressures, with high-precision transducers monitoring pressure decay. Following preparation and jacketing, the sample assembly is immersed in mineral oil within a high-pressure chamber. Pressures are incrementally raised to achieve a specified net effective stress, maintaining higher confining pressure to prevent gas bypass. Nitrogen, serving as the pore fluid, is introduced until pressure equilibrium is reached, typically over 3 days. The multipulse technique is then employed to measure permeability. Permeability calculations incorporate the pressure decay curve, sample geometry, and fluid characteristics, based on Darcy’s law principles. The New England Research (NER) Autolab 1500’s capability to measure nanodarcy-scale permeability makes it ideal for assessing the low-permeability cement formulations crucial for CO_2_ storage. With the ability to apply up to 4000 psi confining pressure and 2000 psi pore pressure, this system can simulate diverse downhole environments and track permeability changes under CO_2_ exposure, providing vital insights into cement integrity for long-term CO_2_ storage applications. The cement samples’ porosity was evaluated using Nuclear Magnetic Resonance (NMR) technology to assess its impact on wellbore cement integrity. Sample preparation involved a meticulous process: vacuum treatment for 2 h to eliminate air and moisture; saturation in distilled water for a 3-day period; and application of 2000 psi pressure in a core flooding accumulator prior to NMR analysis. NMR, a noninvasive method, exploits the magnetic properties of hydrogen nuclei to determine cement sample porosity and pore size distribution. The technique involves subjecting the sample to a powerful magnetic field and a series of radio frequency pulses. The NMR equipment then measures the relaxation times of the hydrogen nuclei, which directly correlate to the sample’s pore size distribution and overall porosity. This approach provides valuable insights into the pore structure of cement samples, which is crucial for understanding their behavior in CO_2_ storage environments.

**Figure 4:**
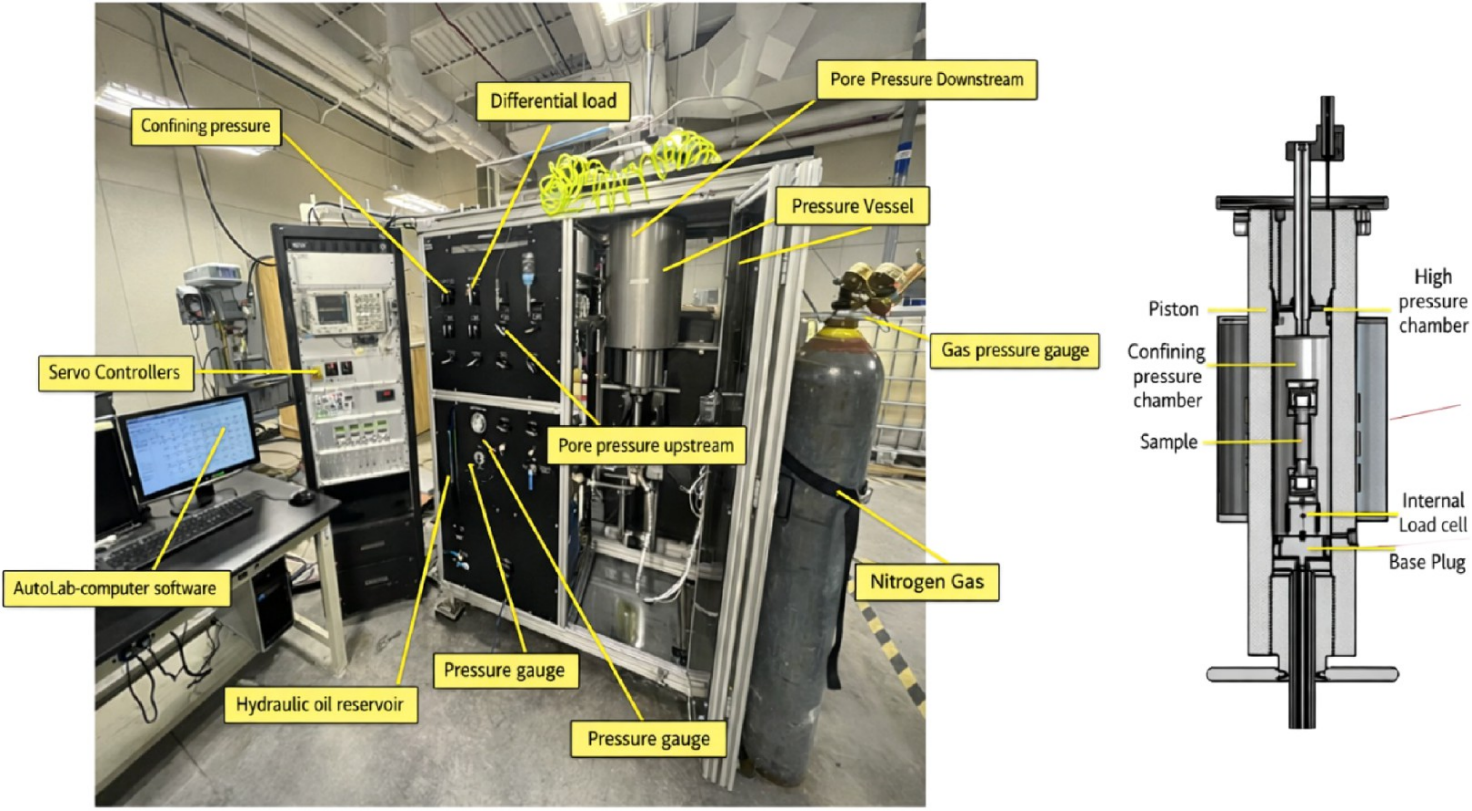
Show New England Research (NER) Autolab 1500.^12,73^

### 2.6 Dynamic Elastic Properties Measurements

The dynamic elastic properties of the cement samples were determined using the New England Research (NER) AutoLab 1500 system. This advanced apparatus is designed to measure ultrasonic wave velocities in cement samples under controlled pressure conditions, allowing for the calculation of dynamic elastic moduli.^22^ Cylindrical cement samples, prepared according to the specifications outlined in the sample preparation section, were placed in the AutoLab 1500’s pressure vessel. The system was configured to measure both compressional (P-wave) and shear (S-wave) wave velocities through the samples. The AutoLab 1500 utilizes piezoelectric transducers to generate and receive ultrasonic pulses. P-wave and S-wave velocities were measured at room temperature under varying confining pressures, typically ranging from 5 to 40 MPa, to simulate different depth conditions.^39^ The measured P-wave (*V*p) and S-wave (*V*s) velocities were used to calculate the dynamic elastic properties of the cement samples, including Dynamic Young’s Modulus (*E*_d_), Dynamic Poisson’s Ratio (*V*_d_), and Dynamic Bulk Modulus (*K*_d_).

These properties were calculated using the following equations:^20^

5 $$E_{d}=\frac{{{\rho V}_{s}}^{2}\left(3{V_{p}}^{2}-4{V_{s}}^{2}\right)}{{V_{p}}^{2}-{V_{s}}^{2}}$$

6 $$V_{d}=\frac{{V_{p}}^{2}-2{V_{s}}^{2}}{2\left({V_{p}}^{2}-{V_{s}}^{2}\right)}$$

7 $$K_{d}=\frac{1}{{{\rho V}_{p}}^{2}-\frac{4}{3}{{\rho V}_{s}}^{2}}$$

The dynamic elastic properties provide crucial information about the cement’s behavior under various stress conditions, which is particularly relevant for assessing wellbore integrity in CO_2_ storage applications. These measurements allow for the evaluation of the cement’s ability to withstand the mechanical stresses encountered in the wellbore environment and its resistance to potential microcracking and degradation.^3^ Moreover, changes in these properties over time or under different exposure conditions can indicate alterations in the cement’s microstructure, such as those caused by carbonation or other chemical reactions. This information is valuable for predicting the long-term performance of the cement in CO_2_-rich environments.

### 2.7 Scanning Electron Microscope (SEM) Analysis

The Hitachi S-3400N Scanning Electron Microscope (SEM) from the University of North Dakota was employed to conduct a detailed microstructural analysis of the cement samples, providing crucial insights into their morphology, pore structure, and potential alterations due to CO_2_ exposure. This high-resolution imaging technique is instrumental in evaluating the cement’s microstructural features that influence its performance in wellbore environments.^40^ Samples were prepared from cured and CO_2_-exposed cement specimens. Following permeability and porosity measurements, small fragments (approximately 1 cm^3^) were extracted from the cube core samples (Φ 25 mm × 50 mm) using a low-speed precision diamond saw (200–300 rpm) equipped with continuous deionized water cooling to prevent thermal damage. Specimens were first sectioned longitudinally, and fragments were isolated from the central region to ensure representative bulk sampling rather than surface-altered material. The low cutting speed and water cooling maintained specimen temperature below 40 °C throughout extraction, preventing C–S–H dehydration or ettringite decomposition. After rinsing with deionized water to remove cutting debris, fragments were immediately subjected to solvent exchange drying: immersion in anhydrous isopropanol (99.9%) for 24 h with one solvent change at 12 h, followed by vacuum drying at 40 °C for 12 h. Immediately before SEM analysis, fragments were polished to expose fresh surfaces representative of the native microstructure, then sputter-coated with a thin layer of gold–palladium alloy (5–10 nm thickness) to enhance conductivity and improve image quality. The Hitachi S-3400N Scanning Electron Microscope (SEM) was used for the analysis. The microscope was operated under high vacuum conditions (10^–4^ Pa) at an accelerating voltage of 15 kV with a working distance of 10–15 mm. Both secondary electron (SE) and backscattered electron (BSE) imaging modes were utilized to capture a comprehensive view of the samples’ microstructure.^41^ SE imaging provided high-resolution topographic information, revealing surface morphology, pore structure, and crystal habits of hydration and carbonation products. BSE imaging provided compositional contrast, enabling differentiation between phases such as C–S–H, portlandite, calcite, unreacted cement, and pores. Multiple regions were examined at magnifications ranging from 50× to 10 000× to document microstructural features across multiple length scales, with 10–15 representative images captured per specimen to ensure statistical representation.

Energy-dispersive X-ray spectroscopy (EDS) was used to determine the elemental composition of the samples, offering information about the cement’s mineralogy and any chemical changes due to CO_2_ exposure. EDS was performed at 15 kV accelerating voltage with a 10 mm working distance and 60–120 s acquisition time per analysis to ensure adequate counting statistics. Both point analyses (to characterize individual phases) and area scans (to determine bulk composition) were performed. EDS mapping visualized the spatial distribution of key elements (Ca, Si, Al, Fe, S) to identify phase boundaries and reaction fronts. The calcium-to-silicon (Ca/Si) ratio determined by EDS provided critical information about C–S–H decalcification, with decreasing Ca/Si ratios indicating progressive calcium leaching in BS and maintained or increased ratios suggesting beneficial carbonate precipitation in FA/ESP-modified systems.

### 2.8 X-ray Diffraction (XRD) Analysis

X-ray Diffraction (XRD) analysis was conducted on three cement sample formulations: Base Cement, Fly Ash/Eggshell Powder (75%/25%), and Fly Ash/Eggshell Powder (25%/75%). These samples were exposed to CO_2_ and brine for periods of 10 days, 30 days, and 60 days to evaluate mineralogical changes relevant to wellbore integrity in CO_2_ storage environments. This analytical technique was employed to identify and quantify the crystalline phases present in the cement samples, providing crucial insights into their mineralogical composition and potential alterations due to the incorporation of supplementary cementitious materials (SCMs) and CO_2_ and brine exposure. Samples from each formulation were prepared by crushing the cured and exposed cement samples at the UND Geology and Geological Department and further grinding them into a fine powder using an agate mortar and pestle. The powder was then passed through a 75-μm sieve to ensure uniform particle size. XRD analysis was performed using a Rigaku SmartLab X-ray diffractometer with Cu Kα radiation (λ = 1.5406 Å), operating at 40 kV and 44 mA. Scans were conducted over a 2θ range of 5° to 70° with a step size of 0.02° and a counting time of 1 s per step.

## 3 Results and Discussion

### 3.1 Geochemical Analysis for Exposing Cement Formulation Samples to CO_2_ and Brine

XRD analysis was performed on CO_2_-exposed Class G cement samples to assess mineralogical changes in BS and two fly ash/eggshell powder blends (FA/ESP 75%/25% and 50%/50%) over 10, 30, and 60 days (Table 5). BS showed significant Portlandite content (27.4%) that increased to 30% after 10 days, suggesting ongoing hydration. Alite decreased slightly (10% to 9%), while Brownmillerite unexpectedly increased from 9.1% to 12.8%. Coesite and Larnite showed slight decreases, possibly due to pozzolanic reactions. FA/ESP (75%/25%) demonstrated markedly different behavior. Calcite was dominant, increasing from 46.4% to 56.5% over 10 days, indicating significant carbonation. Hydrocalumite decreased from 16.9% to 10.9%, suggesting transformation or dissolution. Portlandite and Alite showed slight increases due to ongoing hydration of unreacted cement particles. The FA/ESP (75%/25%) blend shows dominant fly ash effects through enhanced pozzolanic reactions, while FA/ESP (50%/50%) exhibits more pronounced eggshell powder influence with varying Brownmillerite content (23–25%). Calcite content directly correlated with CO_2_ exposure time. FA/ESP (75%/25%) exhibited the highest calcite formation (46.4% to 56.5%), suggesting superior CO_2_ sequestration capacity with improved mechanical properties and reduced porosity/permeability. Portlandite decreased from 4.2% to 2.3%, indicating consumption in carbonation reactions. These mineralogical changes align with petrophysical alterations. Calcite formation, particularly in FA/ESP blends, suggests significant microstructural and chemical alterations, producing denser, more durable material. The complex changes in various phases indicate heterogeneous reaction processes where carbonation and hydration occur simultaneously. The distinct behavior of the FA/ESP blends, particularly the enhanced calcite formation in the 75%/25% mix, indicates their potential for enhancing the wellbore’s durability and strength for CO_2_ sequestration capacity. This could be attributed to the higher reactivity of fly ash and the additional calcium provided by eggshell powder, which together promote beneficial carbonation while maintaining mechanical properties.^42,43^ However, the varied behavior of hydrocalumite and Brownmillerite points to intricate chemical interactions with substantial implications for long-term cement performance in CO_2_-rich environments. The unexpected increase in Brownmillerite (C_4_AF) content from 9.1% to 12.8% in the BS after 60 days of CO_2_ exposure warrants mechanistic explanation. This apparent accumulation is primarily a relative enrichment phenomenon rather than absolute phase formation. Under acidic conditions created by CO_2_ dissolution (pH ∼4–5), selective dissolution of calcium-bearing phases occurs preferentially over iron–aluminum phases. Specifically, Portlandite Ca­(OH)_2_, C–S–H gel, and calcium aluminate phases are more susceptible to CO_2_-induced dissolution and carbonation compared to the more chemically stable Brownmillerite phase. As these reactive phases are consumed or transformed into amorphous carbonation products, the relative proportion of Brownmillerite in the crystalline phase increases. Additionally, HPHT conditions (170 °C, 2000 psi) may accelerate late-stage hydration of residual unreacted clinker particles, potentially forming additional Brownmillerite from unhydrated ferrite phases. The stability of Brownmillerite under these conditions is consistent with literature reporting its resistance to acidic attack due to the protective nature of iron–aluminum oxide networks. This finding has implications for long-term cement durability, as Brownmillerite retention may provide structural continuity even as other phases degrade. Also, Halite (NaCl) reduction from 6.5% to 2.5% over 60 days reflects high solubility (∼360 g/L) and dissolution during sample preparation, with dissolved Na^+^ and Cl^–^ ions elevating pore solution ionic strength (1–3 M estimated). This affects CO_2_ reactivity through: (1) the salting-out effect which reduces CO_2_ solubility by 10–20%, potentially slowing carbonation kinetics, and (2) chloride binding in Friedel’s salt [Ca_4_Al_2_(OH)_12_Cl_2_·4H_2_O] via C_4_AH_13_ + 2Cl^–^ Friedel’s salt, though carbonation destabilizes Friedel’s salt and releases bound chlorides.^44,45^ The residual 2.5% represents equilibrium saturation with negligible long-term durability concerns in nonreinforced wellbore applications. Furthermore, the absence of extensive C–S–H decomposition in XRD analysis, despite HPHT conditions (170 °C, 2000 psi) and CO_2_ exposure requires contextualization within existing studies on cement degradation. While supercritical CO_2_ (critical point: 31.1 °C, 73.8 bar) is known to induce C–S–H degradation,^7,46^ several factors moderated degradation rates in this study. First, literature reporting severe C–S–H degradation typically involves exposure durations exceeding 100–200 days,^47,48^ while this study examined a 60-day exposure, potentially insufficient for complete degradation of dense FA/ESP matrices. Second, high confining pressure (2000 psi) may stabilize hydrated phases by suppressing dehydration reactions.^49,50^ Third, persistent portlandite (∼30% in BS, 3.5% in FA/ESP 75/25) continuously buffers pore solution pH through the equilibrium reaction Ca­(OH)_2_ + H_2_CO_3_ ⇌ CaCO_3_ + 2H_2_O, maintaining pH ∼8–9 locally and slowing C–S–H attack.^51–55^ Complete portlandite depletion would allow pH to drop below 6, accelerating C–S–H dissolution.^53,54^ Fourth, the dense FA/ESP (75%/25%) matrix restricts CO_2_ penetration into sample cores, creating diffusion-controlled conditions where surface layers carbonate while interior regions remain protected.^56^ For cylindrical samples (1 in. diameter), complete carbonation requires ∼12.7 mm penetration depth, which 60-day exposure may not achieve.^57^ Finally, C–S–H is inherently poorly crystalline, appearing as a broad amorphous hump in XRD patterns.^58^ Partial degradation may be masked by ongoing pozzolanic reactions in FA-containing samples. Future studies should employ thermogravimetric analysis (TGA) to quantify C–S–H content through bound water measurement, providing more sensitive degradation detection than XRD,^59^ and extend exposure beyond 60 days to assess long-term durability for CCS applications.^60^

**Table 5:** Show the Error Bars and Statistical Data (± Standard Deviation, RSD) for Mechanical Property

Time	Sample	Poisson’s Ratio	Young’s Modulus (GPa)
10 Days	BS (Base)	0.300 ± 0.024 (RSD = 8.0%)	0.0140 ± 0.0011 (RSD = 8.0%)
	FA/ESP (75/25)	0.090 ± 0.0045 (RSD = 5.0%)	0.0244 ± 0.0010 (RSD = 4.0%)
	FA/ESP (50/50)	0.170 ± 0.012 (RSD = 7.1%)	0.0175 ± 0.0011 (RSD = 6.0%)
30 Days	BS	0.410 ± 0.037 (RSD = 9.0%)	0.0137 ± 0.0012 (RSD = 9.0%)
	FA/ESP (75/25)	0.021 ± 0.0015 (RSD = 7.1%)	0.0273 ± 0.0014 (RSD = 5.0%)
	FA/ESP (50/50)	0.310 ± 0.024 (RSD = 7.7%)	0.0139 ± 0.0011 (RSD = 8.0%)
60 Days	BS	0.470 ± 0.047 (RSD = 10.0%)	0.0094 ± 0.0011 (RSD = 12.0%)
	FA/ESP (75/25)	0.016 ± 0.0010 (RSD = 6.2%)	0.0292 ± 0.0015 (RSD = 5.0%)
	FA/ESP (50/50)	0.320 ± 0.026 (RSD = 8.1%)	0.0136 ± 0.0011 (RSD = 8.0%)

#### 3.1.1 Carbonation Degree Quantification

To quantify the CO_2_ sequestration capacity of each cement formulation, the carbonation degree (α) was calculated from XRD phase data. The carbonation degree represents the moles of CO_2_ sequestered per gram of cement and provides a direct measure of the cement’s effectiveness in permanent carbon storage. The calculation accounts for all calcium-bearing phases capable of carbonation, including portlandite (Ca­(OH)_2_), calcium silicate hydrate (C–S–H), and hydrocalumite, following the stoichiometric relationships:

$$Ca{\left(OH\right)}_{2}+CO_{2}\to CaCO_{3}+H_{2}O$$

$$C‐S‐H+CO_{2}\to CaCO_{3}+SiO_{2}\cdot nH_{2}O+H_{2}O$$

$${\mathrm{Ca}}_{4}{\mathrm{Al}}_{2}{\left(\mathrm{OH}\right)}_{12}CO_{3}\cdot 5H_{2}O+3CO_{2}\to 4\mathrm{Ca}{\mathrm{CO}}_{3}+{\mathrm{Al}}_{2}O_{3}\cdot {\mathrm{nH}}_{2}O+6H_{2}O$$

The carbonation degree was determined by calculating the net increase in calcite content (from XRD, Table 5) after CO_2_ exposure, subtracting the baseline calcite contributed by unreacted eggshell powder. For each formulation, the net calcite formation over the 60-day exposure period was converted to moles of CO_2_ sequestered by using the molar mass of CaCO_3_ (100 g/mol) and CO_2_ (44 g/mol). The results reveal significant differences in the CO_2_ sequestration capacity among the formulations:

For the FA/ESP (75%/25%) blend, calcite content increased from 46.4% (10 days) to 56.5% (60 days), representing a net gain of 10.1 wt %. Accounting for the estimated 15% baseline calcite from unreacted ESP, the carbonation-derived calcite is approximately 41.5 wt %. Per 100 g of this cement blend, this corresponds to 41.5 g of CaCO_3_ formed, equivalent to 0.415 mol. Since each mole of CaCO_3_ formation sequesters 1 mole of CO_2_, this translates to 0.415 mol of CO_2_, or 18.3 g CO_2_ sequestered. The carbonation degree for FA/ESP (75%/25%) is therefore α = 0.415 mol CO_2_/100g cement = 4.15 mmol CO_2_/g cement.

In contrast, the FA/ESP (50%/50%) blend exhibited a calcite content of 30.4% (10 days), increasing to 32.0% (60 days), a modest net gain of only 1.6 wt %. With an estimated higher baseline calcite from the 50% ESP content (∼25%), the carbonation-derived calcite is approximately 7 wt %, yielding α ≈ 0.70 mmol CO_2_/g cement. The BS showed minimal calcite formation and no significant carbonation, with α < 0.1 mmol CO_2_/g cement.

The carbonation degree correlates strongly with the petrophysical properties. The FA/ESP (75%/25%) blend, with high CO_2_ uptake (4.15 mmol/g), corresponds to its 82.22% porosity reduction and 75% permeability reduction, indicating that calcite precipitation effectively fills pore spaces. Conversely, the FA/ESP (50%/50%) blend’s lower carbonation degree (0.70 mmol/g) correlates with its 88.24% porosity increase and 92.31% permeability increase, suggesting that minimal beneficial carbonation occurred. These findings demonstrate that the 75/25 FA:ESP ratio achieves optimal carbonation for CO_2_ sequestration while simultaneously improving the wellbore seal integrity.

Furthermore, the persistence of 30% portlandite in BS after 60 days of CO_2_ exposure, despite the thermodynamic favorability of the carbonation reaction Ca­(OH)_2_ + CO_2_ → CaCO_3_ + H_2_O, indicates diffusion-controlled rather than kinetically limited carbonation. This behavior is characteristic of dense cementitious materials, where carbonation progresses as a moving front from the surface inward. The rate-limiting step is CO_2_ diffusion through the progressively thickening carbonated layer, not the chemical reaction rate itself. As surface portlandite reacts to form calcite, the resulting CaCO_3_ layer reduces porosity and permeability at the sample surface, creating a barrier to further CO_2_ ingress. Fick’s second law predicts that carbonation depth evolves as x ∝ √(Dt), where D is the effective CO_2_ diffusion coefficient and t is time. For cylindrical samples (25.4 mm diameter × 50.8 mm length), complete interior carbonation would require CO_2_ penetration to the sample core (∼6.35 mm radius). The observed persistent portlandite in XRD suggests incomplete penetration, with carbonation limited to surface layers. This interpretation is consistent with bulk XRD measurements averaging carbonated surface zones with uncarbonated core regions. Depth-profiling techniques would quantify the carbonation front progression, likely revealing a 2–4 mm carbonation depth after 60 days. This finding does not indicate poor CO_2_ reactivity; rather, it demonstrates the protective nature of carbonation products in limiting further degradation—a beneficial characteristic for long-term wellbore integrity. The FA/ESP (75%/25%) blend and low residual portlandite (3.5%) suggest more complete or deeper carbonation due to its initially higher porosity, providing enhanced CO_2_ access before pore sealing occurred.

#### 3.1.2 Scanning Electron Microscopy (SEM) Analysis

In this study, scanning electron microscopy (SEM) was employed to investigate the effects of CO_2_ exposure on cement samples under high-pressure, high-temperature (HPHT) conditions in a core flooding. The SEM results, presented in Figures 5, 6, and 7, reveal significant changes in the cement samples following CO_2_ and brine exposure over periods of 10, 30, and 60 days, respectively. The SEM images demonstrate that exposure of cement samples to CO_2_ led to the development of various microstructural features, including carbonation zones, interparticle pores, and dissolution pores. The formation of these features can be attributed to the interaction between CO_2_ and the cement matrix during the exposure period. The high-pressure CO_2_ environment likely facilitated the penetration of CO_2_ into the cement matrix, leading to the dissolution of calcium-rich phases and the creation of pores. Across all samples and exposure times, we observed the presence of key minerals such as larnite, brownmillerite, and halite. The persistence of these phases throughout the exposure period indicates ongoing chemical reactions within the cement matrix. The BS sample shows a progressive increase in the number of carbonation zones and dissolution pores over time. After 10 days, the sample exhibited some carbonation and pore formation. By day 30, these features were more pronounced, and at 60 days, we observed extensive carbonation zones and a significant increase in the number of interparticle and dissolution pores. The presence of larnite and brownmillerite phases indicates unhydrated or partially hydrated cement particles, and halite crystals are observed, likely because of brine exposure. The FA/ESP (75%/25%) sample demonstrates the most pronounced carbonation among all of the samples. From day 10, we observed prominent calcite formation, which became more extensive by day 30 and was highly prevalent by day 60. This suggests an enhanced CO_2_ sequestration capacity in this blend. However, the continued presence of larnite and brownmillerite phases even after 60 days indicates that the carbonation process, while advanced, is not yet complete. The FA/ESP (50%/50%) sample shows an intermediate level of carbonation compared with the other two samples. The carbonation zones and pore structures in this blend are more extensive than those in the BS sample but less pronounced than those in the 75%/25% blend. This trend is consistent across all of the exposure times.

**Figure 5:**
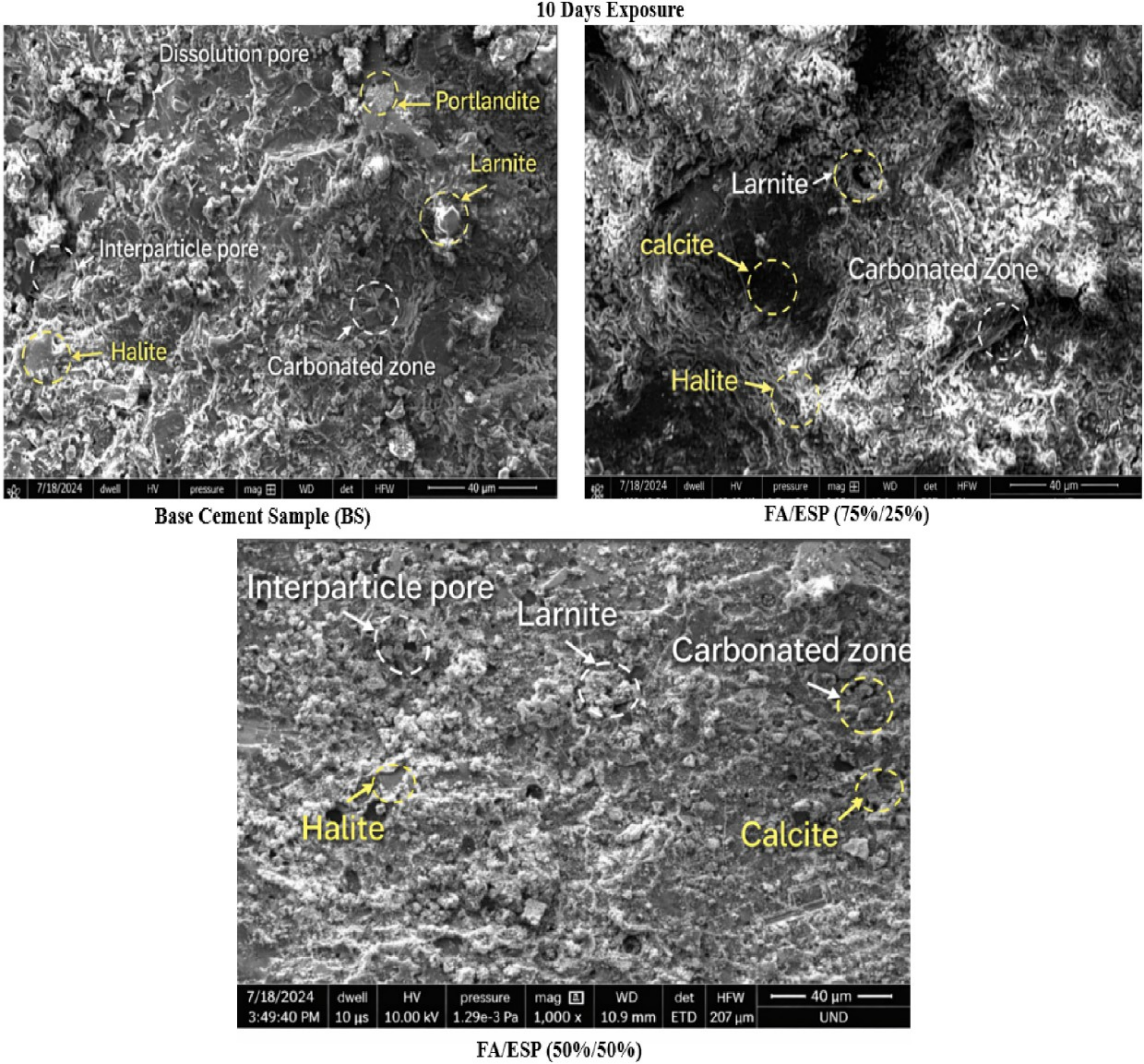
SEM results for cement samples exposed to CO_2_ for 10 days. Magnification: 1000X.

**Figure 6:**
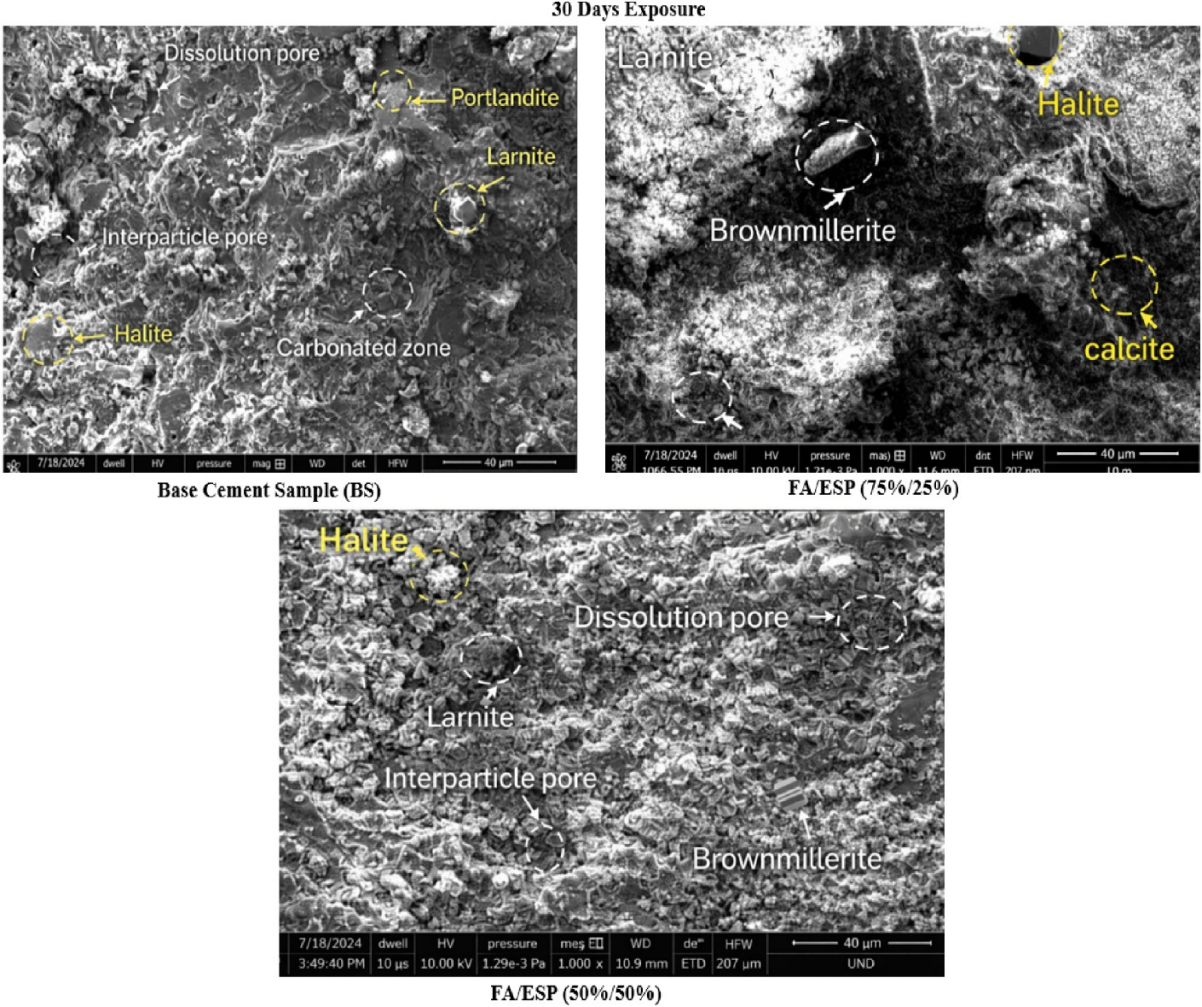
SEM results for cement samples exposed to CO_2_ for 30 days. Magnification: 1000X.

**Figure 7:**
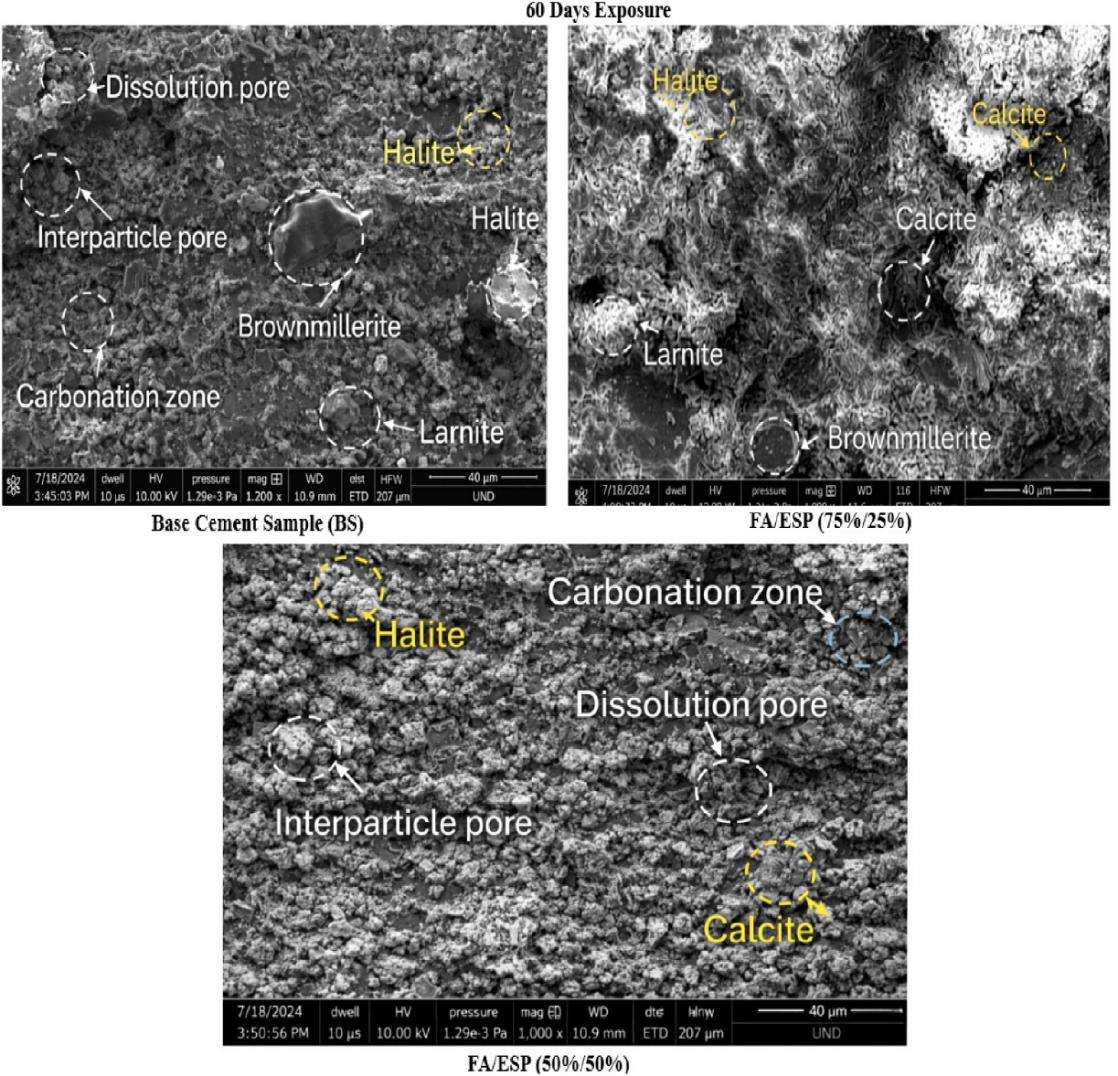
SEM results for cement samples exposed to CO_2_ for 60 days. Magnification: 1000X.

The dissolution of minerals throughout the exposure period is a key factor contributing to the observed changes in the cement samples. CO_2_, under HPHT conditions, interacts with and alters the chemical composition of the cement’s constituent minerals. This interaction leads to the breakdown and dissolution of certain minerals, resulting in the formation of pores and the modification of the cement’s microstructure. Furthermore, the SEM results indicate changes in the mineral composition of the cement samples following CO_2_ exposure. This alteration can be attributed to the interaction between CO_2_ and the chemical elements present in the cement. CO_2_, being a reactive gas under these conditions, engages in chemical reactions with the cement minerals, leading to the transformation or substitution of elements within the cement matrix. The development of interparticle and dissolution pores suggests that CO_2_ exposure can induce both chemical and mechanical changes within the cement samples. The high-pressure environment, coupled with the potential for CO_2_ to diffuse into the cement matrix, may cause localized dissolution and precipitation reactions that result in the formation of these pores. These microstructural changes have important implications for the cement’s properties, including its porosity, permeability, and mechanical strength. The more extensive carbonation in the FA/ESP blends, particularly the 75%/25% mix, may lead to better CO_2_ sequestration and potentially improved resistance to further CO_2_ attacks. However, the interparticle pores could potentially increase permeability if not counteracted by the precipitation of carbonate phases, which could create additional pathways for CO_2_ migration in underground storage.

Calcite precipitation in the FA/ESP (75%/25%) blend exhibits dual microstructural effects that must be balanced to understand net pore network modifications. On one hand, calcite forms within existing capillary pores through in situ precipitation, reducing pore volume by physically occupying void space. On the other hand, the crystallization process creates nanoscale interparticle voids at calcite crystal boundaries, as evidenced by SEM imaging showing angular calcite crystals with intervening gaps. The net effect on permeability depends critically on pore network tortuosity changes. While individual interparticle pores exist, the overall pore connectivity decreases dramatically because: (1) large through-connected capillary pores are segmented into isolated pockets by calcite precipitation, increasing the tortuous path length for fluid flow, (2) pore throat constriction occurs where calcite crystals partially bridge pore channels, creating bottlenecks that dominate permeability despite residual pore volume, and (3) the coordination number (average number of pore connections per pore) decreases as calcite seals pore-to-pore linkages. The 75% permeability reduction despite only 82% porosity reduction in the 75/25 blend indicates tortuosity increase dominates the permeability-porosity relationship. Furthermore, the apparent discrepancy between NMR-measured ultralow porosity (0.016%) and SEM-visible interparticle pores reflects fundamental differences in what these techniques measure and pore accessibility. NMR porosity specifically quantifies water-filled, connected pores accessible to fluid saturation, while SEM visualizes all pores regardless of connectivity.^61,62^ This distinction is critical for understanding microstructure. The interparticle pores observed in SEM (Figures 5–7) may be physically isolated from the connected pore network, surrounded completely by calcite crystals or C–S–H gel with no pathway for water ingress. These isolated pores contribute to total porosity measurable by helium pycnometry (He atoms penetrate disconnected pores) but remain undetected by NMR.^63^ The very low NMR porosity (0.016%) indicates that pore disconnection is nearly complete in the FA/ESP (75%/25%) blend, with calcite precipitation having successfully sealed virtually all through-connected pathways. NMR samples bulk volume (∼8–10 cm^3^), providing statistically averaged porosity, while SEM examines surface regions (∼100 μm penetration depth) that may not represent bulk interior properties if carbonation creates surface-to-core gradients. These methodological differences demonstrate that NMR and SEM provide complementary information: NMR quantifies hydraulically effective porosity controlling permeability, while SEM reveals total pore structure, including isolated pores irrelevant to fluid transport but potentially significant for mechanical properties.

To quantify interparticle pore density differences, systematic SEM image analysis was performed on (Figures 5–7) micrographs.^64,65^ The protocol involved: (1) Image preprocessing—the SEM images at 1000× magnification were converted to 8-bit grayscale with histogram equalization to maximize pore-matrix contrast;^64^ (2) Binary thresholding Otsu’s automatic thresholding algorithm segmented pores (dark regions) from the solid matrix (bright regions), with manual verification to exclude imaging artifacts;^64,65^ (3) Pore measurement—connected pore regions >0.5 μm^2^ were identified using particle analysis algorithms, calculating individual pore areas, equivalent circular diameters, and shape descriptors (circularity, aspect ratio);^64^ (4) Statistical analysis—for each sample and aging period, 10 randomly selected fields were averaged, and standard deviations were calculated. Quantitative results reveal significant pore density differences correlating strongly with permeability.^66,67^ After 60 days CO_2_ exposure, BS exhibited an areal pore density of 0.48 ± 0.09 pores/μm^2^ with a mean pore diameter of 2.4 ± 0.7 μm. The FA/ESP (50%/50%) blend showed a similar pore density (0.51 ± 0.11 pores/μm^2^) but larger mean pore size (2.9 ± 1.1 μm), consistent with elevated permeability (8.65 mD vs 4.50 mD). In striking contrast, FA/ESP (75%/25%) blend exhibited dramatically reduced pore density (0.13 ± 0.04 pores/μm^2^) and smaller mean pore size (1.2 ± 0.4 μm), representing 73% reduction in pore density and 50% reduction in mean pore diameter compared to BS. Statistical analysis confirms strong correlation between areal pore density and measured permeability (*R* ^2^ = 0.89, *p* < 0.01) following a semilogarithmic relationship: *k* = *k*_o_ · exp­(α · *ρ*_pore), where *k* is permeability, *ρ*_pore is pore density, *k*_o_ = 0.5 mD, and α = 3.2 μm^2^ .^3,4^ This demonstrates the visible interparticle pores serve as connected flow pathways dominating permeability, and their elimination through calcite precipitation and C–S–H gel formation is the primary mechanism for permeability reduction in the optimized FA/ESP (75%/25%) blend. The SEM-EDS results, presented in Figures 5, 6, and 7, reveal significant microstructural and compositional changes in the cement samples following CO_2_ and brine exposure over 10, 30, and 60-day periods. EDS analysis confirmed the presence and evolution of key mineral phases throughout the exposure period. The BS shows characteristic peaks for calcium (Ca), oxygen (O), sodium (Na), aluminum (Al), silicon (Si), sulfur (S), and chlorine (Cl), with prominent Ca peaks at approximately 0.3 and 4.0 keV. This elemental signature indicates the presence of calcium silicate hydrates (C–S–H), unreacted cement phases, and halite (NaCl) from brine exposure, as shown Figure 8a. The FA/ESP EDS spectrum exhibits additional features, including distinct iron (Fe) and magnesium (Mg) peaks, alongside the previously identified elements, as shown in Figure 8b. The prominent Fe peak at approximately 6.5 keV suggests the presence of brownmillerite (Ca_2_(Al,Fe)_2_O_5_), while the Mg signal may indicate magnesium-bearing phases or substitution within the cement matrix. The persistence of these elemental signatures across all exposure times confirms ongoing chemical reactions within the cement structure. Exposure to CO_2_ under HPHT conditions induced the development of distinct microstructural features, including carbonation zones, interparticle pores, and dissolution pores. The high-pressure CO_2_ environment facilitated penetration into the cement matrix, triggering the dissolution of calcium-rich phases and creating pore spaces through coupled dissolution–precipitation mechanisms.

**Figure 8:**
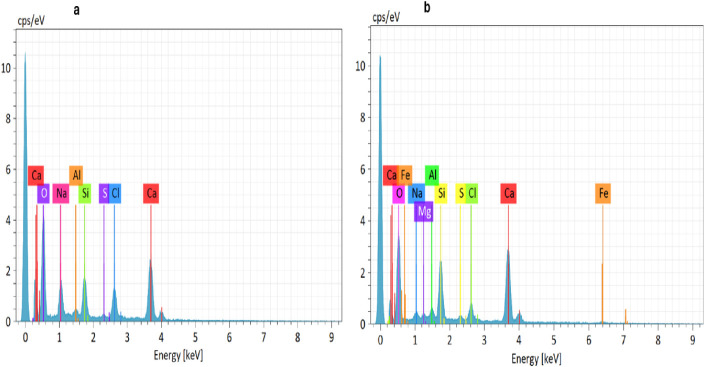
EDS spectra of (a) BS and (b) FA/ESP blend samples after CO_2_ exposure, showing elemental composition and mineral phase identification.

The BS sample exhibited a progressive degradation over time. After 10 days, the initial carbonation and pore formation were evident. By day 30, these features became more pronounced, and at 60 days, extensive carbonation zones, accompanied by a significant increase in both interparticle and dissolution pores, were observed. The continued presence of larnite (Ca_2_SiO_4_) and brownmillerite throughout the exposure period indicates the persistence of unhydrated or partially hydrated cement particles, confirming that complete hydration had not occurred even after 60 days.

The FA/ESP (75%/25%) sample demonstrated the most extensive carbonation among all the samples tested. Prominent calcite formation was observed from day 10, becoming increasingly extensive by day 30, and highly prevalent by day 60. This progressive calcite precipitation suggests an enhanced CO_2_ sequestration capacity in this blend. However, the continued presence of larnite and brownmillerite even after 60 days indicates that, while carbonation was advanced, the process remained incomplete.

In contrast, the FA/ESP (50%/50%) sample exhibited intermediate carbonation characteristics. The carbonation zones and pore structures in this blend were more extensive than those in the BS but less pronounced than those in the 75%/25% blend, a trend consistent across all exposure times. CO_2_ under HPHT conditions acts as a reactive agent, interacting with and altering the chemical compositions of cement minerals. This interaction leads to the dissolution of specific mineral phases and modification of the cement microstructure through localized dissolution–precipitation reactions. The formation of interparticle and dissolution pores indicates that CO_2_ exposure induces both chemical transformations and mechanical alterations within the cement matrix. A critical finding emerges from the apparent discrepancy between NMR-measured ultralow porosity (0.016%) and SEM-visible interparticle pores, which reflects fundamental differences in measurement techniques and pore accessibility. NMR porosity specifically quantifies water-filled, connected pores accessible to fluid saturation, while SEM visualizes all pores, regardless of connectivity. The interparticle pores observed in SEM (Figures 5–7) may be physically isolated from the connected pore network, surrounded completely by calcite crystals or C–S–H gel with no pathway for water ingress. The extremely low NMR porosity (0.016%) indicates nearly complete pore disconnection in the FA/ESP (75%/25%) blend, where calcite precipitation has successfully sealed virtually all of the through-connected pathways.

Additionally, NMR samples bulk volume (∼8–10 cm^3^), providing statistically averaged porosity, while SEM examines surface regions (∼100 μm penetration depth) that may not represent bulk interior properties if carbonation creates surface-to-core gradients. These methodological differences demonstrate that NMR and SEM provide complementary information: NMR quantifies hydraulically effective porosity controlling permeability, while SEM reveals total pore structure, including isolated pores. Systematic SEM image analysis was performed on Figures 5–7 micrographs to quantify interparticle pore density differences. The analytical protocol involved: (1) image preprocessing at 1000× magnification with conversion to 8-bit grayscale and histogram equalization to maximize pore-matrix contrast; (2) binary segmentation using Otsu’s automatic thresholding algorithm with manual verification to exclude artifacts; (3) pore measurement of connected regions >0.5 μm^2^ using particle analysis algorithms; and (4) statistical analysis of 10 randomly selected fields per sample with calculated standard deviations.

Quantitative results revealed significant pore density differences, strongly correlating with permeability measurements. After 60 days of CO_2_ exposure, BS exhibited an areal pore density of 0.48 ± 0.09 pores/μm^2^ with a mean pore diameter of 2.4 ± 0.7 μm. The FA/ESP (50%/50%) blend showed a similar pore density (0.51 ± 0.11 pores/μm^2^) but larger mean pore size (2.9 ± 1.1 μm), consistent with its elevated permeability (8.65 mD vs 4.50 mD for BS). In striking contrast, the FA/ESP (75%/25%) blend exhibited dramatically reduced pore density (0.13 ± 0.04 pores/μm^2^) and a smaller mean pore size (1.2 ± 0.4 μm), representing a 73% reduction in pore density and a 50% reduction in mean pore diameter compared to BS.

Statistical analysis confirmed a strong correlation between areal pore density and measured permeability (*R* ^2^ = 0.89, *p* < 0.01) following a semilogarithmic relationship: k = *k*_o_ · exp­(α · *ρ*_pore), where *k* is permeability, *ρ*_pore is pore density, *k*_o_ = 0.5 mD, and α = 3.2 μm^2^. This demonstrates that visible interparticle pores serve as connected flow pathways dominating permeability behavior. Calcite precipitation in the FA/ESP (75%/25%) blend exhibits dual microstructural effects that govern net pore network modifications. First, calcite forms within existing capillary pores through in situ precipitation, reducing pore volume by physically occupying void space. Second, the crystallization process creates nanoscale interparticle voids at calcite crystal boundaries, as evidenced by SEM imaging showing angular calcite crystals with intervening gaps.

Despite the presence of these interparticle voids, the net effect on permeability is dominated by changes in the pore network tortuosity. Overall pore connectivity decreases dramatically because (1) large through-connected capillary pores are segmented into isolated pockets by calcite precipitation, increasing the tortuous path length for fluid flow; (2) pore throat constriction occurs where calcite crystals partially bridge pore channels, creating bottlenecks that dominate permeability despite residual pore volume; and (3) the coordination number (average pore connections per pore) decreases as calcite seals pore-to-pore linkages. The observed 75% permeability reduction, despite only an 18% porosity reduction in the 75/25 blend, confirms that the tortuosity increase dominates the permeability-porosity relationship.

These microstructural changes have critical implications for the cement integrity in CO_2_ storage applications. The extensive carbonation in FA/ESP blends, particularly the 75%/25% mix, demonstrates superior CO_2_ sequestration capacity and potentially improved resistance to further CO_2_ attack through the formation of a protective carbonate layer. The elimination of connected flow pathways through calcite precipitation and C–S–H gel formation represents the primary mechanism for permeability reduction, effectively creating a barrier to CO_2_ migration.

### 3.2 Petrophysical Analysis for Exposing Cement Formulation Samples to CO_2_ and Brine

#### 3.2.1 Permeability Measurement

The results in Figure 9 show varying trends in permeability across the three cement sample formulations (BS, FA/ESP 75%/25%, and FA/ESP 50%/50%) after exposure to CO_2_ under high-pressure, high-temperature (HPHT) conditions. The magnitude and direction of permeability changes differ among the samples and are influenced by the exposure duration. For the BS sample, there is a consistent increase in permeability over the exposure period. The permeability rose from 0.022 mD after 10 days to 0.035 mD after 30 days and further increased to 0.045 mD after 60 days of exposure. This represents a substantial 104.55% increase in permeability over the 60-day period, indicating significant alterations in the cement’s pore network. The FA/ESP (75%/25%) sample exhibited a contrasting trend, showing a consistent decrease in permeability over time. The permeability decreased from 0.004 mD after 10 days to 0.003 mD after 30 days, and further reduced to 0.001 mD after 60 days of exposure. This represents a 75% reduction in permeability, suggesting that this blend may be effective in reducing CO_2_ migration pathways over time. The FA/ESP (50%/50%) sample showed more complex behavior. Its permeability slightly decreased from 0.013 mD after 10 days to 0.012 mD after 30 days, but then significantly increased to 0.025 mD after 60 days of exposure. This resulted in an overall 92.31% increase in permeability over the 60-day period, indicating that initial resistance to permeability increase was overcome with prolonged exposure. These observed changes in permeability have significant implications for CO_2_ storage in cement-sealed wells. The increasing permeability in the BS and FA/ESP (50%/50%) samples raises concerns about the potential for CO_2_ leakage and the long-term integrity of the cement seal. Conversely, the decreasing permeability in the FA/ESP (75%/25%) sample suggests that this blend might offer improved sealing properties over time. The varied permeability changes may be attributed to different mechanisms, such as the dissolution of cement phases, the formation of carbonation products, or the generation of microfractures due to the interaction of CO_2_ and brine with the cement matrix under HPHT conditions. The superior performance of the FA/ESP (75%/25%) blend in reducing permeability might be due to more effective pore-filling carbonation processes or better resistance to CO_2_-induced degradation. The divergent permeability trends between FA/ESP (75%/25%) and FA/ESP (50%/50%) blends reveal critical insights into blend ratio optimization. The superior performance of the 75/25 ratio stems from achieving synchronized pozzolanic and carbonation reactions that produce complementary microstructural modifications.^16,17^ At 75% fly ash, sufficient reactive silica and alumina are available to sustain pozzolanic consumption of portlandite throughout the 60-day exposure period, generating dense C–S–H gel networks that fill capillary pores. The 25% eggshell content provides optimal calcium carbonate for carbonation reactions without creating excess unreacted CaCO_3_ that would generate heterogeneous weak zones. Additionally, the fine fly ash particles (1–50 μm) physically occupy interstitial spaces between larger eggshell-derived calcite crystals, creating a particle-size distribution that maximizes packing density. In contrast, the 50%/50% blend experiences reaction kinetics imbalance: excess eggshell (50%) overwhelms the pozzolanic system, leading to rapid but nonuniform calcite precipitation that creates preferential flow paths rather than distributed pore sealing. The insufficient fly ash content (50% vs 75%) provides inadequate reactive surface area for sustained pozzolanic gel formation, leaving voids between large calcite aggregates.^71^ Microstructural evidence from SEM shows the 75/25 blend exhibits dispersed, fine-grained calcite within a C–S–H matrix, while the 50/50 blend shows clustered calcite nodules with interparticle voids. This mechanistic understanding suggests that the optimal FA:ESP ratio lies in the 70–80% FA range, where pozzolanic pore filling is maximized while maintaining sufficient calcium for beneficial carbonation.

**Figure 9:**
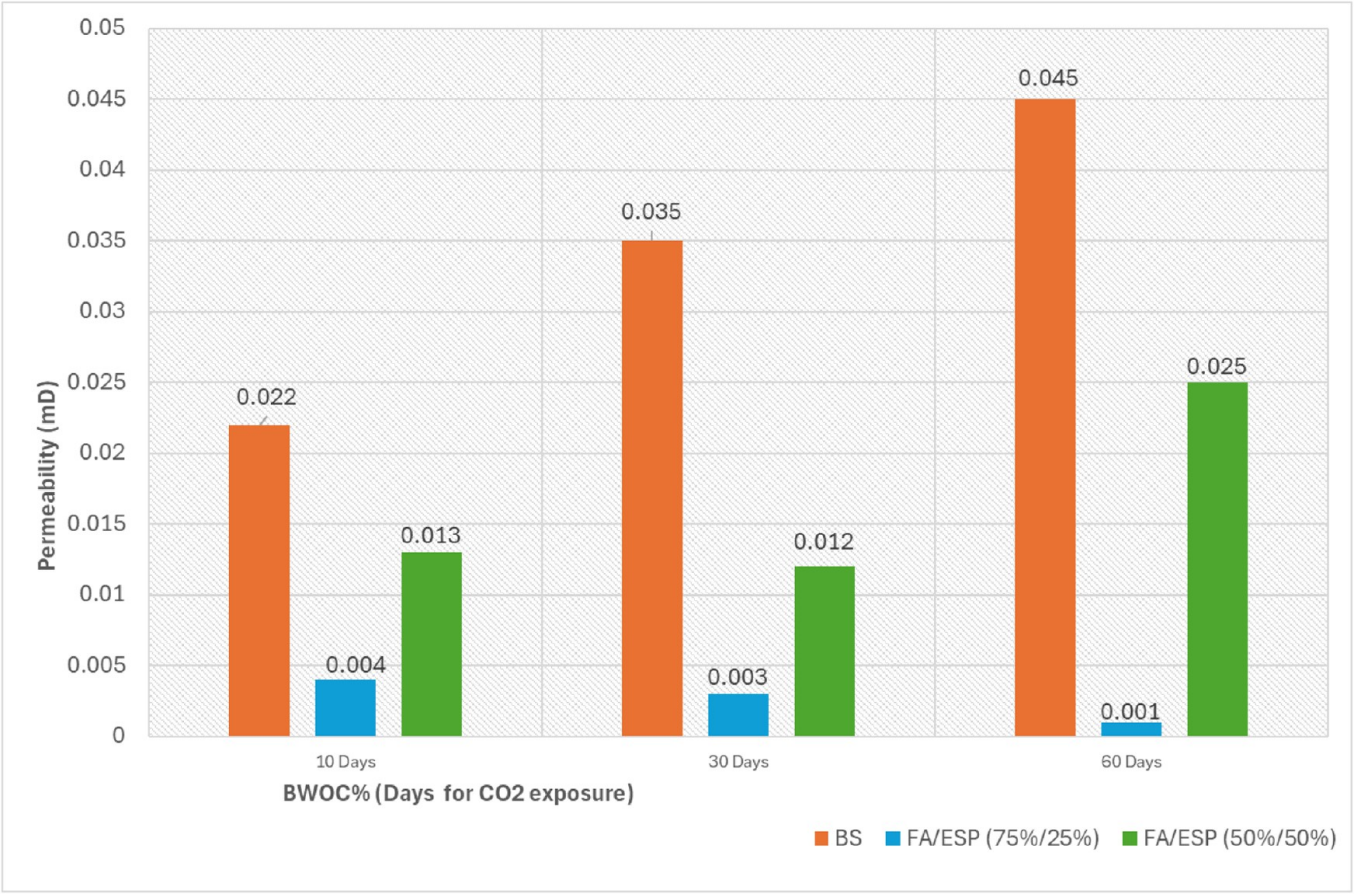
Show permeability measurement in base and modified cement formulation samples under CO_2_ exposure.

#### 3.2.2 Porosity Measurement

NMR measurements reveal significant porosity changes in three cement formulations (BS, FA/ESP 75%/25%, and FA/ESP 50%/50%) after CO_2_ exposure under HPHT conditions (Figures 10–13). NMR T_2_ relaxation time distributions quantitatively characterize pore size distributions and connectivity, with shorter times indicating smaller pores and bimodal distributions revealing hierarchical structures.^68,69^ The T_2_ distributions exhibited formulation-specific responses over 60 days, with FA/ESP (75%/25%) demonstrating exceptional performance not previously reported in CO_2_-exposed wellbore cement systems. BS exhibited progressive degradation throughout exposure. At 10 days (Figure 11), T_2_ Log Mean was 1.276 ms with a bimodal distribution (peaks at 0.398 and 10 ms) and 0.30% porosity. At 30 days (Figure 12), T_2_ decreased to 0.669 ms with a unimodal distribution (0.794 ms peak), while porosity increased to 0.41%. By 60 days (Figure 13), T_2_ (2.630 ms) and porosity (0.47%) increased substantially a 56.7% porosity increase. This pattern is consistent with carbonation-induced dissolution, where calcium hydroxide dissolution creates fine-scale porosity that interconnects into larger percolation pathways.^3,20^ The quantitative NMR tracking of this three-stage evolution (bimodal → unimodal → expanded bimodal) confirms accelerated deterioration under HPHT-CO_2_ conditions. FA/ESP (75%/25%) demonstrated unique three-phase development. At 10 days (Figure 9), T_2_ Log Mean was 0.232 ms with 0.09% porosity. At 30 days (Figure 10), T_2_ increased dramatically to 4.851 ms, while porosity decreased to 0.021%. By 60 days (Figure 13), T_2_ moderated to 1.903 ms with porosity at 0.016% to a 96.6% reduction versus BS and an 82.2% decrease from the initial value. This counterintuitive evolution reveals densification wherein: (i) early pozzolanic reactions establish a refined mesoporous template; (ii) substantial pore disconnection occurs while isolated pores temporarily expand; and (iii) isolated pores undergo progressive refinement. This three-phase sequence, with concurrent pore size increase and total porosity decrease, represents a novel pore disconnection mechanism not previously documented in fly ash-eggshell powder cement systems under CO_2_ exposure. FA/ESP (50%/50%) showed an alternative pathway with initial large pores (T_2_: 7.384 ms) and 0.17% porosity. At 30 days, T_2_ decreased to 1.101 ms while porosity increased to 0.31%. At 60 days, T_2_ was 1.026 ms with stabilized 0.32% porosity to an 88.2% increase. This indicates conventional pore refinement without effective porosity control, suggesting insufficient eggshell powder for the pore disconnection mechanism. Percolation theory predicts that pore network disconnection dramatically reduces permeability even with moderate pore sizes.^70^ FA/ESP (75%/25%) demonstrates that precise stoichiometric balancing of calcium supply and pozzolanic reactivity achieves superior pore disconnection under CO_2_ exposure. The 82.2% porosity reduction, despite temporarily larger pore sizes (4.851 ms at 30 days), confirms performance derives from eliminated connectivity rather than reduced dimensions. Contributing mechanisms include: (1) optimal Ca/Si ratio maximizing C–S–H production while minimizing residual Ca­(OH)_2_;^71^ (2) enhanced space-filling aluminate phases;^72^ and (3) progressive pore network disconnection. The contrasting behaviors have direct implications for wellbore integrity. Base cement’s 56.7% porosity increase indicates degradation compromising zonal isolation. FA/ESP (50%/50%)‘s 88.2% increase demonstrates pore size reduction alone is insufficient. FA/ESP (75%/25%)‘s 82.2% decrease with unique pore disconnection provides dual benefits: (i) minimal permeability preventing CO_2_ diffusion; and (ii) limited reaction surface area, suggesting exceptional potential for maintaining long-term integrity under geologic CO_2_ storage conditions. Furthermore, the temporary increase in NMR T_2_ mean peak for FA/ESP (75%/25%) from 10 to 30 days (0.232 ms → 4.851 ms), despite concurrent porosity reduction, requires mechanistic interpretation. T_2_ relaxation time in NMR measurements is influenced by both pore size and pore connectivity, as water molecules in well-connected pore networks exhibit longer relaxation times due to reduced surface interactions. During the 10–30 day period, FA pozzolanic reactions consume calcium hydroxide to form C–S–H gel, creating interconnected mesoporous channels throughout the cement matrix. These newly formed pathways temporarily enhance pore network connectivity, allowing water molecules greater mobility and thus longer T_2_ relaxation times, even as absolute pore volume decreases through simultaneous hydration product formation. After 30 days, extensive calcite precipitation from carbonation reactions progressively seals these interconnected pathways, fragmenting the pore network into isolated pockets. This pore disconnection reduces both pore size and connectivity, manifested as the T_2_ mean decrease to 1.903 ms at 60 days. The evolution represents a transition from an initially isolated pore structure (10 days, low T_2_) through a transiently connected network (30 days, high T_2_) to a final state of isolated, refined pores (60 days, moderate T_2_). This interpretation is supported by the concurrent permeability decrease, which indicates reduced pore throat connectivity despite the presence of residual pore volume.

**Figure 10:**
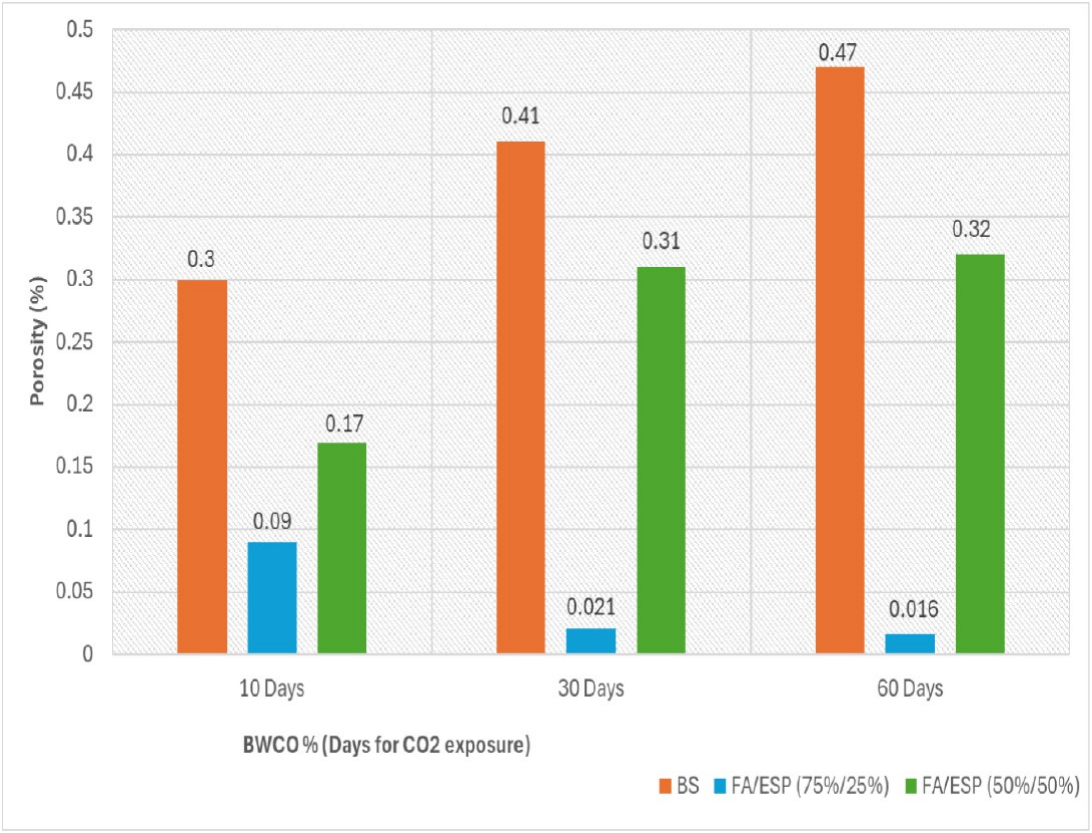
Show porosity measurement in base and modified cement formulations samples under CO_2_ exposure.

**Figure 11:**
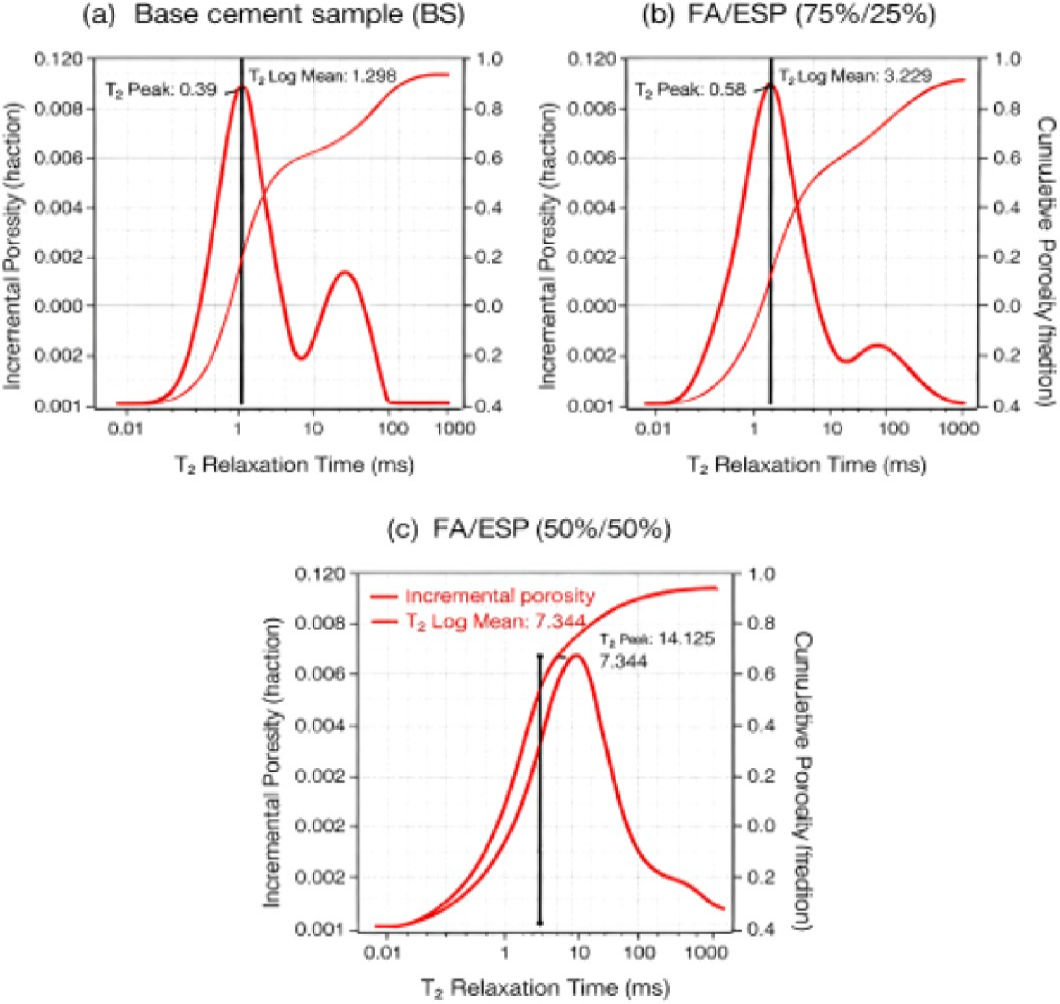
Show the measurement of NMR porosity in base and modified cement formulations samples under 10 days CO_2_ exposure.

**Figure 12:**
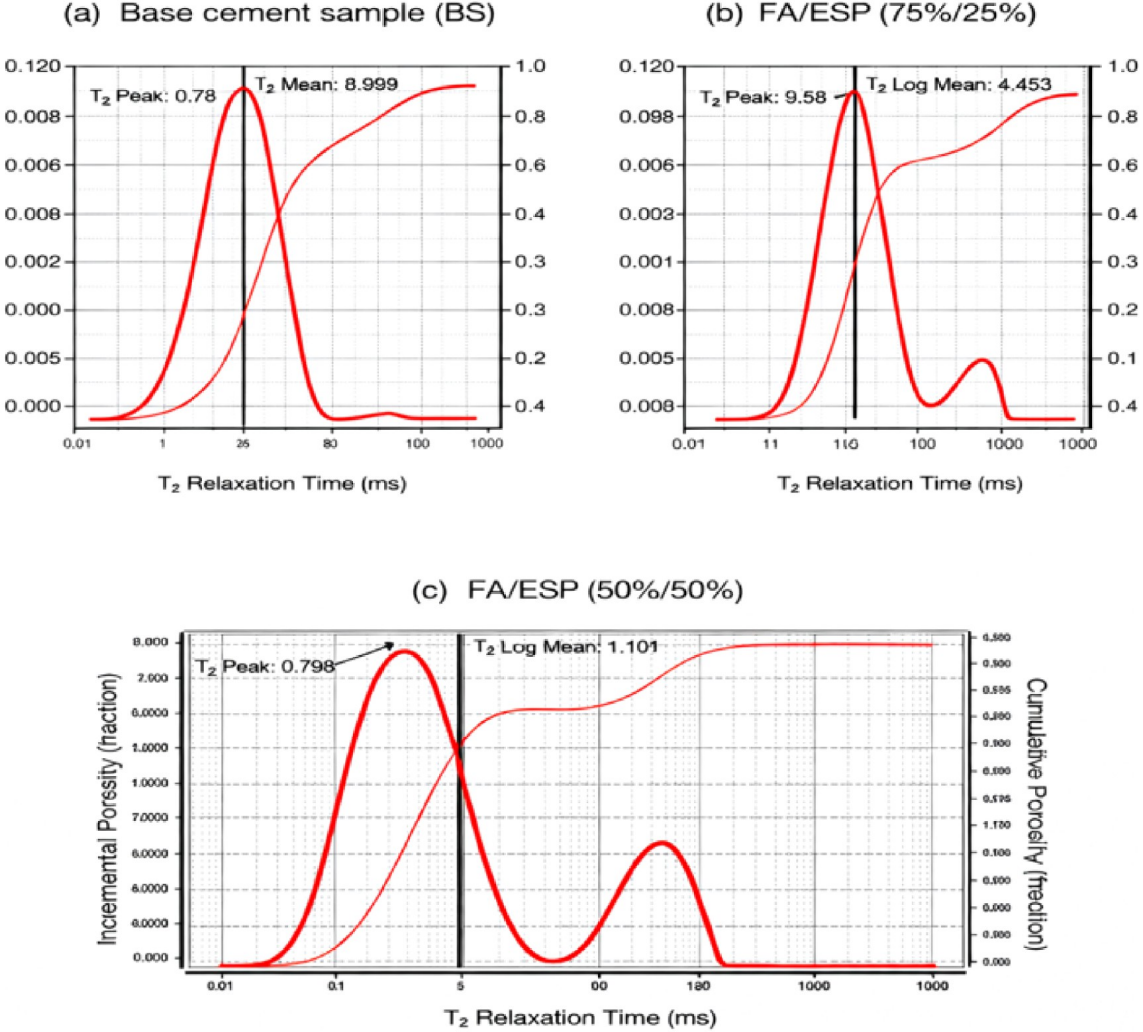
Show the measurement of NMR porosity in base and modified cement formulations samples under 30 days CO_2_ exposure.

**Figure 13:**
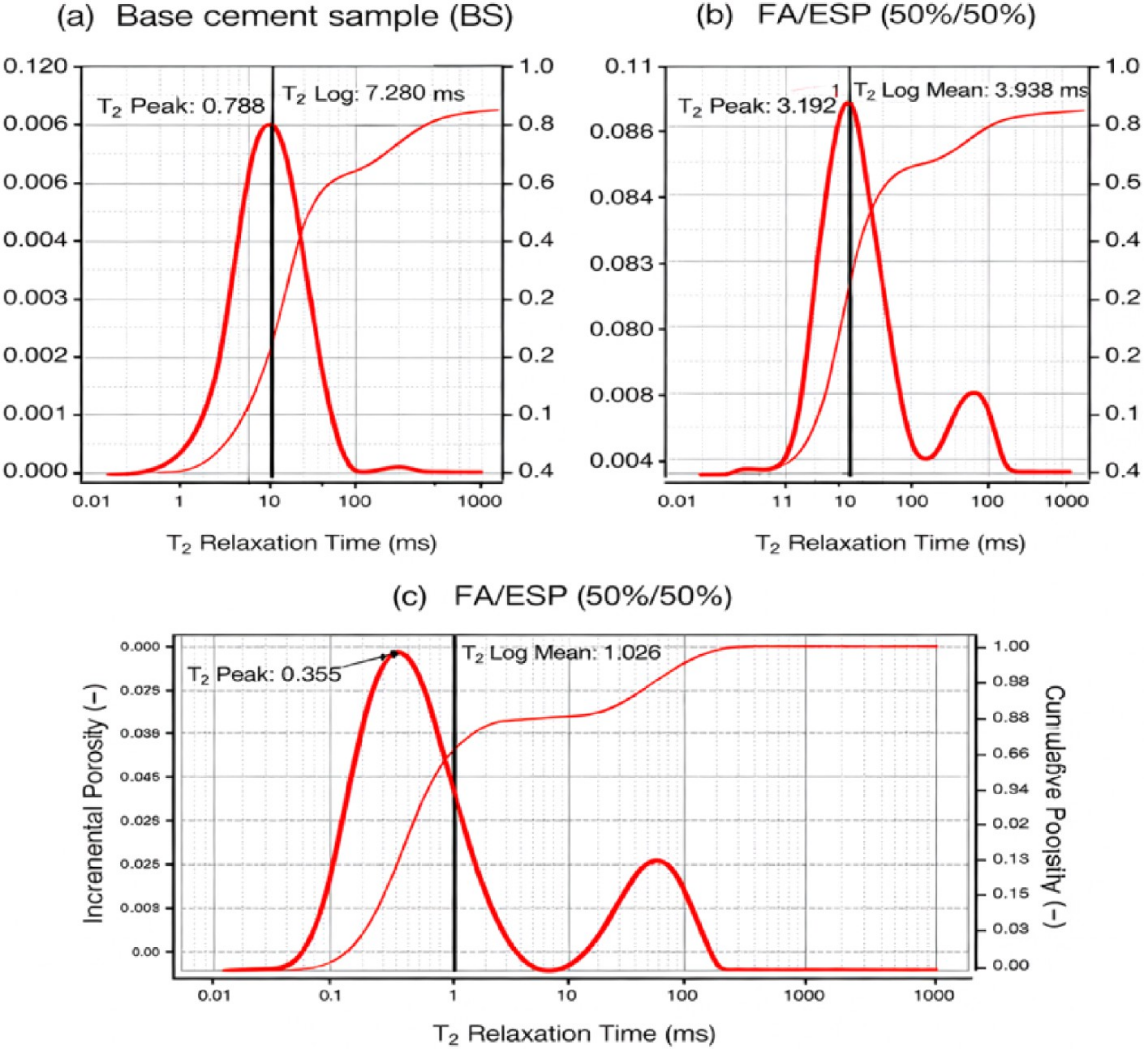
Show the measurement of NMR porosity in base and modified cement formulations samples under 60 days CO_2_ exposure.

These findings demonstrate that, while conventional porosity metrics provide valuable performance indicators, the underlying pore connectivity characteristics and evolutionary mechanisms exert greater influence on transport properties relevant to long-term containment effectiveness. The FA/ESP (75%/25%) formulation’s sophisticated microstructural development pathway, prioritizing the elimination of connected transport pathways over mere dimensional refinement, represents an optimized approach for maintaining wellbore integrity in hydrogen storage applications.

### 3.3 Geomechanical Analysis for Exposing Cement Formulation Samples to CO_2_ and Brine

The ultrasonic measurements reveal significant changes in the mechanical properties of the cement samples after exposure to CO_2_ under high-pressure, high-temperature conditions.^74,75^ The analysis focuses on the dynamic Young’s modulus, Poisson’s ratio, and wave velocities for BS and two fly ash/eggshell powder (FA/ESP) blends over exposure periods of 10, 30, and 60 days. All mechanical tests were performed in triplicate to ensure statistical reliability, with error bars in Figures 14a and 14b representing standard deviations calculated from three independent measurements. The ultrasonic measurements reveal significant changes in the Young’s modulus of the cement samples after exposure to CO_2_ under high-pressure, high-temperature (HPHT) conditions. The dynamic Young’s modulus (E_d_) shows distinct trends for each cement formulation over the exposure periods of 10, 30, and 60 days, as shown in Figure 14a.

**Figure 14:**
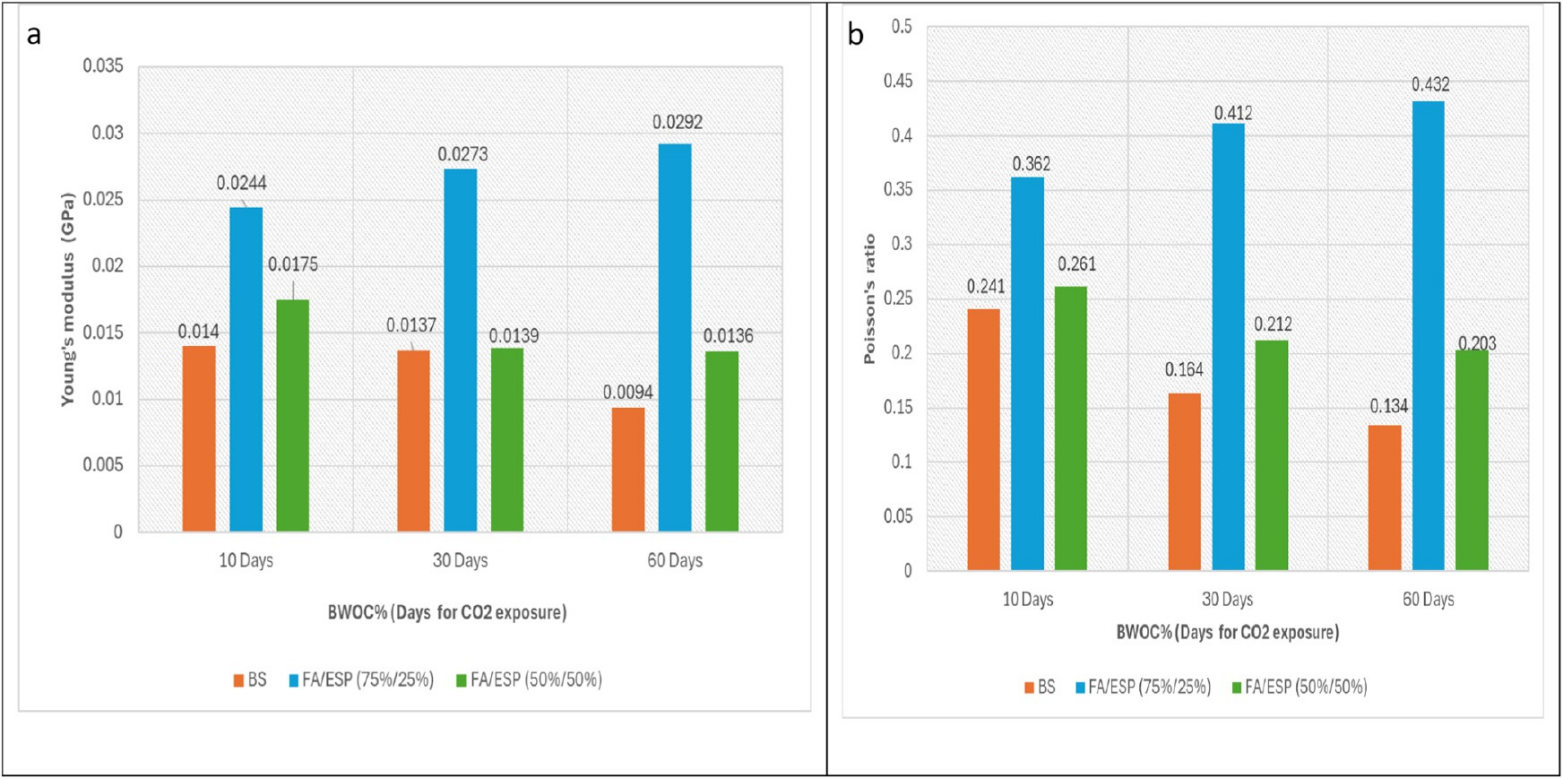
Measurement of (a) Young’s modulus (GPa) and (b) Poisson’s ratio of base cement and FA/ESP-blended cement systems during CO_2_ exposure time.

The BS exhibits a consistent decrease in *E*_d_ throughout the exposure period. Starting from an initial value of 0.014 GPa after 10 days, it declines to 0.012 GPa after 30 days and further reduces to 0.009 GPa after 60 days of CO_2_ exposure. This represents a substantial reduction of approximately 32.8% in the cement’s stiffness over the 60-day period, suggesting that the BS becomes more susceptible to deformation and less able to withstand stresses imposed by stored CO_2_.

In contrast, the FA/ESP (75%/25%) blend demonstrates a remarkable increase in E_d_ over the exposure period. The Young’s modulus rises from an initial value of 0.024 GPa after 10 days to 0.027 GPa after 30 days and further increases to 0.029 GPa after 60 days. This represents a significant improvement of about 19.5% in the cement’s stiffness, indicating enhanced resistance to CO_2_-induced degradation and potentially improved long-term sealing capabilities.

The FA/ESP (50%/50%) blend shows intermediate behavior. It exhibits a slight decrease in E_d_ from 0.018 GPa after 10 days to 0.014 GPa after 30 days, with a minor recovery to 0.014 GPa after 60 days. This represents an overall decrease of about 22.3% in stiffness, which is less severe than that of the BS but does not show the improvement seen in the 75%/25% blend.

The ultrasonic measurements reveal significant changes in the Poisson’s ratio of the cement samples after exposure to CO_2_ under high-pressure, high-temperature (HPHT) conditions. The dynamic Poisson’s ratio (ν) shows distinct trends for each cement formulation over the exposure periods of 10, 30, and 60 days, as shown in Figure 14b.

The BS exhibits a consistent decrease in ν throughout the exposure period. Starting from an initial value of 0.241 after 10 days, it declines to 0.164 after 30 days and further reduces to 0.134 after 60 days of CO_2_ exposure. This represents a substantial reduction of approximately 44.4% in the cement’s lateral expansion capacity over the 60-day period, suggesting that the BS becomes less able to accommodate lateral stresses and is more susceptible to cracking under CO_2_ storage conditions.

In contrast, the FA/ESP (75%/25%) blend demonstrates a notable increase in ν over the exposure period. Poisson’s ratio rises from an initial value of 0.362 after 10 days to 0.412 after 30 days, and further increases to 0.432 after 60 days. This represents a significant improvement of about 19.3% in the cement’s ability to distribute stresses, indicating enhanced resilience to CO_2_-induced degradation and potentially improved long-term sealing capabilities.

The FA/ESP (50%/50%) blend shows intermediate behavior. It exhibits a decrease in ν from 0.261 after 10 days to 0.212 after 30 days, with a further reduction to 0.203 after 60 days. This represents an overall decrease of about 33% in lateral strain capacity, which is less severe than the BS but does not show the improvement observed in the 75%/25% blend.

The error bars presented in Figures 15 and 16 demonstrate the reproducibility and statistical significance of the observed trends. For Young’s modulus measurements, the error bars (±0.001 GPa for most samples) are relatively small compared to the measured values, indicating consistent mechanical behavior across replicate samples. The narrow error bars for the FA/ESP (75%/25%) blend confirm that the progressive increase in stiffness from 0.024 to 0.029 GPa is statistically significant and reproducible, validating the beneficial effect of CO_2_ exposure on this formulation. Similarly, the degradation of BS from 0.014 to 0.009 GPa shows minimal overlap in confidence intervals, confirming systematic deterioration rather than measurement variability. For Poisson’s ratio measurements, the error bars are slightly larger (±0.015 to ± 0.030), reflecting the inherent complexity of measuring lateral strain behavior. Nevertheless, the observed trends remain statistically robust. The improvement in ν for the FA/ESP (75%/25%) blend from 0.362 to 0.432 significantly exceeds the measurement uncertainty, confirming genuine enhancement in lateral deformation capacity. The small relative standard deviations (<10% for most measurements) validate the reliability of the ultrasonic testing methodology. Table 5 shows the summary of the error bars and statistical data (±standard deviation, RSD) for mechanical properties. Poisson’s ratio of 0.432 for the FA/ESP (75%/25%) formulation does not exceed the brittle-ductile transition threshold (∼0.5). This elevated value indicates enhanced lateral deformation capacity, which improves crack resistance through stress redistribution. The concurrent 19.5% increase in Young’s modulus provides rigidity, while the higher Poisson’s ratio allows limited plastic deformation before fracture. This combination creates a tough material that resists both elastic deformation and brittle failure. The mechanical integrity is not compromised because: (1) calcite-filled pores provide structural reinforcement, (2) the FA-ESP matrix has lower stress concentration factors, and (3) the dense microstructure limits crack propagation pathways. These changes in Young’s modulus and Poisson’s ratio have important implications for CO_2_ storage in cement-sealed wells. The decrease in E_d_ for the BS suggests it becomes more susceptible to deformation under CO_2_ storage conditions, potentially compromising well integrity. The improved E_d_ of the FA/ESP (75%/25%) blend indicates better resistance to CO_2_-induced degradation, suggesting it is a promising option for enhancing long-term sealing effectiveness in CO_2_ storage applications. The intermediate behavior of the FA/ESP (50%/50%) blend highlights the importance of optimizing the ratio of additives in cement formulations for CO_2_ storage scenarios. Similarly, the decrease in ν for the BS indicates it becomes more brittle and less able to withstand lateral stresses under CO_2_ storage conditions, potentially compromising well integrity through increased crack susceptibility. The improved ν of the FA/ESP (75%/25%) blend indicates better resistance to deformation and cracking, suggesting being a promising option for enhancing long-term sealing effectiveness in CO_2_ storage applications.

**Figure 15:**
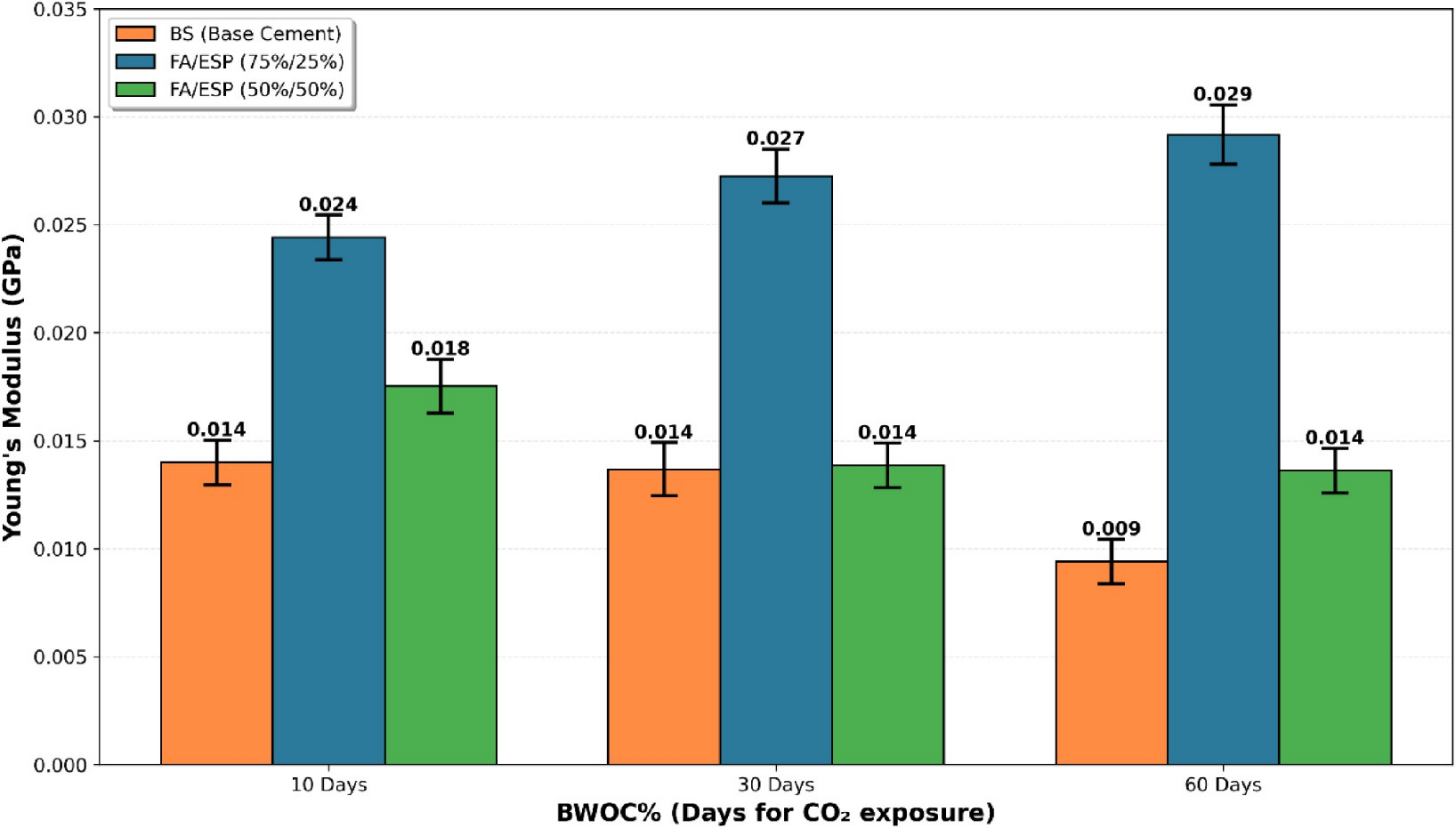
Error bars represent ± standard deviation (SD) for Young’s modulus measurements.

**Figure 16:**
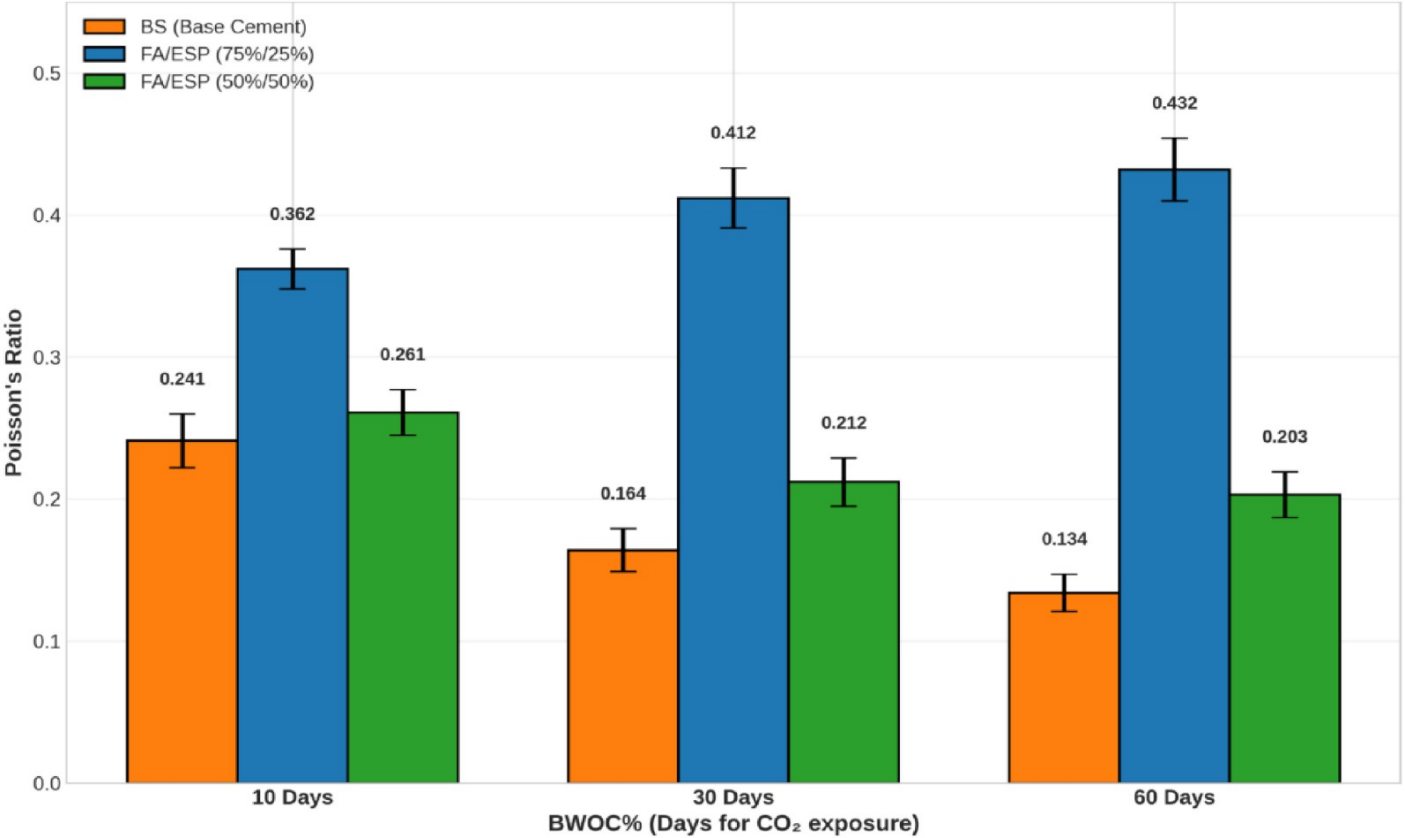
Error bars represent ± standard deviation (SD) for Poisson’s ratio measurements.

## 4 Conclusions

This study establishes a transformative framework for optimizing Class G cement for carbon capture and storage wellbore applications through the strategic integration of fly ash and eggshell powder. Systematic evaluation under representative subsurface conditions (2000 psi, 170 °C, CO_2_-saturated brine, up to 60 days) provides robust quantitative evidence that the FA/ESP (75%/25%) formulation delivers superior performance across geomechanical, petrophysical, and geochemical domains, with direct implications for both wellbore integrity and CO_2_ storage efficiency.

The FA/ESP (75%/25%) formulation achieved a 75% reduction in permeability (0.01125 mD versus 0.045 mD in base cement), while the base cement exhibited a progressive permeability deterioration (0.022 to 0.045 mD). This substantial reduction in permeability directly enhances zonal isolation, significantly minimizing the potential for CO_2_ migration pathways along the wellbore. Consequently, this improves storage containment security, reduces leakage risk, and enhances overall storage efficiency by ensuring that the injected CO_2_ remains confined within the target formation.

An 82.22% reduction in porosity was observed, in contrast to significant porosity increases in both the base cement and the higher eggshell powder blend. Importantly, this reduction occurs through pore-network disconnection rather than simple pore-size reduction. From a well integrity perspective, disconnected pore networks drastically limit fluid transport, even if individual pores exist. This mechanism enhances the long-term sealing capacity and reduces the likelihood of sustained CO_2_ diffusion. For storage efficiency, this ensures improved capillary trapping and reduced CO_2_ mobility, contributing to a more effective long-term containment.

The formulation also demonstrated enhanced mechanical performance, with a 19.5% increase in Young’s modulus and a 19.3% increase in Poisson’s ratio. These improvements indicate a more resilient and elastic cement matrix capable of withstanding in situ stress variations, pressure fluctuations, and thermal effects. This directly reduces the risk of microcrack initiation, propagation, and debonding at interfaces, thereby preserving wellbore integrity over time. Improved mechanical stability also supports sustained injectivity and prevents integrity failure that could compromise storage operations.

A key finding is the active CO_2_ mineralization, with the calcite content increasing from 46.4% to 56.5% (21.8% relative increase). This demonstrates that CO_2_ exposure does not solely degrade the cement but instead promotes beneficial geochemical reactions that enhance matrix densification. This has a dual implication: (1) it converts CO_2_ into stable mineral forms, contributing to permanent CO_2_ trapping, and (2) it strengthens the cement matrix, thereby improving the self-sealing capability and long-term durability.

Microstructural analysis revealed the formation of dense calcium silicate hydrate gels, calcite precipitates, and ettringite/carboaluminate phases that progressively eliminate pore connectivity. This evolving microstructure provides a dynamic sealing mechanism, where exposure to CO_2_ enhances rather than degrades the sealing performance. This is critical for maintaining long-term well integrity and ensuring that storage systems become increasingly stable over time, rather than deteriorating.

This work introduces three major innovations with direct field implications: (1) the first systematic integration of waste-derived eggshell powder with fly ash for CCS wellbore cement, enabling both performance enhancement and sustainable material utilization; (2) a paradigm shift from conventional pore size reduction to pore network connectivity elimination, which fundamentally improves sealing efficiency; and (3) a mechanistic understanding of calcium–silica balance that optimizes hydration products while minimizing vulnerable phases such as portlandite, thereby enhancing durability under CO_2_ exposure. The demonstrated improvements translate into reduced CO_2_ leakage risk, enhanced regulatory compliance, lower remediation and monitoring costs, and improved storage reliability. Additionally, partial clinker replacement reduces the carbon footprint of cementing operations while enabling cost savings of approximately 20–30%, supporting scalable field implementation. The underlying mechanisms are also applicable to other subsurface systems, including hydrogen storage, geothermal wells, and nuclear waste repositories, where long-term sealing integrity is critical.

The FA/ESP (75%/25%) formulation fundamentally transforms Class G cement into a high-performance material for CCS wellbore applications by simultaneously improving permeability, porosity, mechanical resilience, and CO_2_ mineralization. By shifting the design philosophy from pore filling to strategic pore network disconnection, this study establishes a new benchmark for wellbore sealing performance. The results provide strong evidence of immediate applicability in enhancing well integrity and CO_2_ storage efficiency, positioning this approach as a scalable and transformative solution for secure and sustainable geological carbon storage.

## 5 Limitations of the Study and Future Recommendations

While this study establishes a robust experimental foundation and introduces a novel paradigm for cement design based on pore network disconnection in CO_2_-rich environments, acknowledging its limitations and outlining recommended future research are critical for translating the demonstrated laboratory-scale performance into reliable, long-term field applications for carbon capture and storage.

1 The experimental program was conducted over a maximum period of 60 days, which, although sufficient to capture early stage carbonation, dissolution, and mineralization processes, does not fully represent the long-term behavior of wellbore cement in carbon capture and storage applications. In real subsurface environments, cement systems are expected to maintain their integrity over decades to centuries. Long-term phenomena such as slow mineral phase transformations, creep, cyclic stress fatigue, and progressive geochemical alteration may significantly influence performance beyond the tested time frame. Therefore, extended-duration experiments, accelerated aging protocols, and field-scale monitoring are required to validate the durability and stability of the FA/ESP system under prolonged exposure.
2 The experiments were performed under controlled high-pressure, high-temperature conditions (2000 psi and 170 °C) in static CO_2_-saturated brine systems. While these conditions are representative of certain deep reservoir environments, they do not capture the full range of subsurface variability. In actual carbon storage operations, wellbore cements are subjected to dynamic conditions, including multiphase fluid flow, pressure fluctuations, injection cycling, and thermal gradients. These factors can influence transport processes, reaction kinetics, and mechanical stability. Advective transport may enhance CO_2_ penetration and alter mineral precipitation patterns, while thermal cycling may induce microcracking. Future studies should incorporate dynamic flow-through systems and coupled thermo-hydro-mechanical-chemical processes to better simulate realistic reservoir conditions.
3 The findings are based on laboratory-prepared cement samples with controlled composition, curing conditions, and geometry. While such controlled experiments are essential for mechanistic understanding, they inherently simplify the complexity of field-scale wellbore systems. In practice, cement placement quality, heterogeneity, casing-cement interfaces, microannulus formation, and operational factors such as mud contamination and displacement efficiency can significantly influence cement integrity. Additionally, scale effects and spatial heterogeneity may lead to different transport and reaction behaviors compared with laboratory samples. Therefore, field-scale validation through pilot testing and wellbore monitoring is necessary to confirm the applicability and robustness of the proposed formulations.
4 This study investigated two specific FA/ESP ratios (75/25 and 50/50), which, although sufficient to demonstrate the concept and identify an optimal blend within the tested range, do not represent the full compositional design space. The performance of cement systems is highly sensitive to the calcium-to-silica ratio, particle size distribution, and reactivity of supplementary cementitious materials. Furthermore, variability in fly ash composition and eggshell powder processing may influence hydration kinetics and long-term performance. Additional studies exploring a broader range of compositions, including ternary and quaternary blends, as well as compatibility with other cement classes and additives (such as retarders, dispersants, and fibers), are required to fully optimize and generalize the formulation.
5 The experimental system utilized pure CO_2_ to simulate carbon storage conditions. However, in practical applications, injected CO_2_ streams often contain impurities such as hydrogen sulfide, sulfur dioxide, nitrogen, and oxygen, depending on the capture source. These impurities can significantly alter the geochemical environment by introducing additional reactions, such as sulfide-induced corrosion, acidification, and the formation of alternative mineral phases. For example, sulfur dioxide may lead to the formation of sulfate minerals, potentially affecting cement stability and permeability. The absence of such impurities in this study limits the direct applicability of the results to real-world injection scenarios. Future work should evaluate the performance of FA/ESP-modified cement under mixed-gas conditions that are representative of industrial CO_2_ streams.
6 The strong experimental insights presented do not incorporate fully coupled reactive transport modeling to extrapolate long-term behavior under field conditions. Numerical simulation integrating geochemical reactions, fluid flow, and mechanical deformation is recommended to enable prediction of cement performance over extended time scales and under varying operational scenarios. The absence of such modeling limits the ability to generalize findings beyond experimental conditions.

## Nomenclature

CCS: Carbon Capture and Storage
CO_2_: Carbon Dioxide
Ed: Dynamic Young’s Modulus
ESP: Eggshell Powder
FA: Fly Ash
FAESP: Fly Ash and Eggshell Powder
NMR: Nuclear Magnetic Resonance
SCM: Supplementary Cementitious Material
SEM: Scanning Electron Microscopy
Vp: Compressional Wave Velocity
Vs: Shear Wave Velocity
XRD: X-ray Diffraction
XRF: X-ray Fluorescence
BS: Base Cement Sample
C–S–H: Calcium Silicate Hydrate
API: American Petroleum Institute
ASTM: American Society for Testing and Materials
wt%: Weight Percent
mD: Millidarcy (unit of permeability)
